# Effects of the Momentum project on postpartum family planning norms and behaviors among married and unmarried adolescent and young first-time mothers in Kinshasa: A quasi-experimental study

**DOI:** 10.1371/journal.pone.0300342

**Published:** 2024-03-28

**Authors:** Anastasia J. Gage, Francine E. Wood, Rianne Gay, Pierre Akilimali

**Affiliations:** 1 Department of International Health and Sustainable Development, School of Public Health and Tropical Medicine, Tulane University, New Orleans, Los Angeles, United States of America; 2 Department of Medicine, Center on Gender Equity and Health, University of California, San Diego, La Jolla, California, United States of America; 3 Tulane International, LLC, Kinshasa, Democratic Republic of the Congo; 4 Department of Biostatistics and Epidemiology, Kinshasa School of Public Health, University of Kinshasa, Kinshasa, Democratic Republic of the Congo; University of Salamanca, SPAIN

## Abstract

This study evaluated the effect of Momentum–an integrated family planning, maternal and newborn health, and nutrition intervention–on postpartum family planning norms and behaviors among ever married and never-married first-time mothers age 15–24 in Kinshasa, Democratic Republic of the Congo. Using data collected in 2018 and 2020, we conducted an intent-to-treat analysis among 1,927 first-time mothers who were about six-months pregnant at enrollment. Difference-in-differences models were run for panel data and treatment effects models with inverse-probability weighting for endline-only outcomes. Average treatment effects (ATE) were estimated. Momentum had positive effects on partner discussion of family planning in the early postpartum period (ever married 15–19: ATE = 0.179, 95% CI = 0.098, 0.261; never married 15–19: ATE = 0.131, 95% CI = 0.029, 0.232; ever married 20–24: ATE = 0.233, 95% CI = 0.164, 0.302; never married 20–24: ATE = 0.241, 95% CI = 0.121, 0.362) and discussion with a health worker, and on obtaining a contraceptive method in the early postpartum period, except among never married adolescents. Among adolescents, intervention effects on modern contraceptive use within 12 months of childbirth/pregnancy loss were larger for the never married (ATE = 0.251, 95% CI = 0.122, 0.380) than the ever married (ATE = 0.114, 95% CI = 0.020, 0.208). Full intervention exposure had consistently larger effects on contraceptive behaviors than partial exposure, except among ever married adolescents. Momentum had no effect on normative expectations about postpartum family planning use among adolescents, and on descriptive norms and personal agency among those who were never married. Results for normative outcomes and personal agency underscored the intersectionality between young maternal age and marital status. Future programs should improve personal agency and foster normative change in support of postpartum family planning uptake and tailor interventions to different age and marital status subsets of first-time mothers.

## Introduction

Although the global adolescent birth rate declined from 64.5 births to 42.5 births per 1,000 women age 15–19 between 2000 and 2021, levels remain unacceptably high in sub-Saharan Africa. At 109 births per 1,000 women age 15–19, the adolescent birth rate in Democratic Republic of the Congo (DRC) in 2017–2018 was more than double the global rate [1]. Most pregnancies to women aged 15–19 in the DRC were unintended, about 80%, and about half ended in abortions, many of which were and continue to be unsafe [2]. Repeat pregnancies, having more than one child as an adolescent, are common and one in four Congolese adolescent girls have birth intervals of less than 24 months [3]. Levels of unmet need for contraception are unacceptably high and, in 2018, were higher among currently married adolescents age 15–17 (44%) than among those age 18–19 (30%) and 20–24 (32%) [1]. More than half of all unmarried Congolese adolescents age 15–19 had an unmet need for contraception, with the estimate as high as 66% among 15-17-year-olds [1].

Adolescent childbearing has negative medical and social consequences for both mothers and infants. The risks of delayed and inadequate prenatal care, eclampsia, puerperal endometritis, systemic infections, complications of high blood pressure, anemia, poor weight gain, premature birth, low birth weight, obstructed labor, obstetric fistula, and maternal and infant death are higher among pregnant women age 15–19 than among those age 20–24 [4]. Pregnancy is most challenging socially when the adolescent is unmarried or when teenage childbearing is not common in the adolescents’ geographic area of residence or sociocultural group. Teenage pregnancy may jeopardize girls’ education and employment opportunities and increase their likelihood of living in poverty, especially if they are from socioeconomically disadvantaged backgrounds. Unmarried adolescent girls who are pregnant or mothers may face stigma and rejection from parents, male partners, peers, and community members, contributing to social isolation, constantly changing and unstable living arrangements, feelings of failure and powerlessness, and heightened risks of stress, depression, hopelessness, despair, low self-esteem, and suicidal ideation and attempts [5,6].

Preventing unintended pregnancy and unmet need for contraception among adolescents and young adults is critical for their health and wellbeing and for achieving the Sustainable Development Goals related to good health and wellbeing, education, and gender equality and women’s empowerment. However, adolescents face a myriad of challenges in accessing and using contraception. Fear of side effects, myths and misconceptions, cultural and gender norms, partner disapproval, religious prohibitions, and lack of autonomy or agency may impede adolescents’ access to and demand for contraceptives [7–9]. Logistic barriers often include transport costs, inconvenient clinic hours, and the financial cost of care. Adolescents may also face health facility-level barriers such as the lack of adolescent-friendly health services [9], confidentiality concerns, provision of inadequate information on contraception and side effects, refusal to provide certain contraceptive methods to young people, assumptions about whether an adolescent needs contraception, negative provider attitudes, and lack of provider training on long-acting reversable contraception [7,10–12]. These challenges are amplified among unmarried and/or socially disadvantaged adolescent girls and young adult women.

Recognizing the challenges young people face in accessing and using modern contraception, many governments and international donors have invested in programs that address adolescent girls’ and young women’s access to and use of family planning (FP) services. Existing programs vary in their evaluation designs, focus on first-time parents versus parents with higher order births, inclusion of unmarried mothers age 15–19, and intervention approach and duration. A systematic review by Norton et al. [13] identified five broad categories of interventions with high-quality evaluations and statistically significant impact on contraceptive continuation for at least two years, rapid-repeat pregnancy, and birth rates among adolescents: (1) provision of contraceptive services, monitoring of contraceptive use and provision of contraceptive education to partners and families; (2) postpartum counseling and contraceptives provided soon after delivery; (3) pregnancy and contraceptive use planning; (4) community-based social and behavior change communication; and (5) activities that provide motivation, mentoring, and goal setting. Of the 14 high-quality evaluations considered by Norton et al. [13], none were conducted in sub-Saharan Africa.

Emerging evidence of intervention impact on FP in sub-Saharan Africa appears limited to studies focusing on all women of reproductive age. One study that utilized propensity score matching to construct comparison groups from recent Demographic and Health Surveys (DHS) found that exposure to FP messages increased contraceptive use, with the impact being strongest in Central Africa [14]. A cluster randomized control trial conducted in Ghana among adolescents 13–19 years old in Senior High Schools found that an educational intervention guided by the Health Belief Model significantly improved sexual abstinence and knowledge of pregnancy prevention [15]. Among young women who were currently using a modern method of contraception, one study found home visits combined with group education to have significant effects on receipt of FP counseling, informed choice among those from the poorest households, current use of implants, and method satisfaction [16]. Although many community-based interventions have been implemented to create enabling environments for and shift social norms to support adolescents’ use of modern contraceptives, few of these interventions have been directly linked to FP service delivery [17]. Therefore, there is poor understanding of the extent to which and how social and behavior change interventions in the broader community can change adolescent girls’ and young women’s contraceptive use.

This study evaluated the effectiveness of Momentum, a Bill & Melinda Gates Foundation-funded project designed to increase postpartum family planning (PPFP) uptake, improve care seeking and maternal and newborn health (MNH) practices, and foster more gender-equitable behaviors and attitudes among first-time mothers (FTMs) and their male partners in Kinshasa, the capital city of DRC. We sought to determine whether the project’s interventions had significant effects on PPFP-related norms and behaviors among ever married and never married FTMs age 15–24. We also examined whether the project’s effects on PPFP-related outcomes were significant among both adolescent girls (age 15–19) and young adult women (age 20–24). Previous analyses of the same data set demonstrated the effect of Momentum on increased PPFP-related knowledge, perceived norms, personal agency, partner discussion, and modern contraceptive use [18]. In an analysis of contraceptive users, project effect was detected on receipt of FP counseling, obtaining the current contraceptive method from a community-based health worker, informed choice, and current use of implants versus other modern methods [16]. However, these overall estimates may mask disparities in project effectiveness by FTMs’ age and marital status.

From a health equity perspective, disaggregating the project effects by age and marital status is important for several reasons. Teenage mothers and unmarried mothers are vulnerable groups whose reproductive health issues often stir up controversy. Unmarried mothers, particularly adolescents, may face social exclusion and rejection by their families, school expulsion, the loss of their career aspirations, and unique challenges accessing health care and support during and after pregnancy [19,20]. Unmarried mothers often experience difficult socioeconomic circumstances compared to married mothers [21] and may have a high risk for rapid repeat pregnancies due to insufficient knowledge of FP methods, perceived barriers to contraceptive use and an inability to negotiate sex, compounded by parental reluctance to take daughters who were unmarried mothers to FP clinics [22]. The present analysis contributes to expanding the evidence base on adolescent contraceptive use and promoting a broader understanding of disparities in the effectiveness of PPFP interventions for FTMs. It is hoped that the results of the analysis can inform improvements to the design and delivery of interventions to meet the specific needs of young FTMs depending on their age group and marital status.

## Materials and methods

### Study design

The Momentum pilot study employed a quasi-experimental non-equivalent group design using a pre-test-post-test comparison to evaluate the effects of the project on FP, maternal and child health (MCH), and nutrition outcomes that were measured at baseline and/or endline. The study was conducted in three intervention health zones (Kingasani, Lemba, and Matete) and three comparison health zones (Bumbu, Ndjili and Masina I). The intervention targeted 15-24-year-old nulliparous women who were approximately six months pregnant at baseline and their male partners, and a standard of care comparison group. Participants were followed-up for 16 months. The baseline survey was conducted from September to November 2018 and the post-intervention endline survey, from May to August 2020. A pretested structured interviewer-administered questionnaire was used to collect data on sociodemographic characteristics of participants, FP, antenatal care, delivery and postnatal care, exposure to Momentum interventions, fertility preferences, gender relations, and child health.

### Intervention

The Momentum project was built on a model tested in 2015 of using nursing students to deliver contraception at the community level in Kinshasa [23]. Momentum extended the latter model to (1) offer an integrated package of counseling, services, and referrals for FP, MNH, and nutrition; (2) target not only young mothers and their male partners but also key household influencers; and (3) address the gender dynamics that could constrain FTM’s agency for decision-making. The Momentum interventions were delivered from September 2018 to January 2020 and comprised:

a. Training of 150 nursing students (75 males and 75 females recruited from 11 nursing schools) and 20 project supervisors in the gender-integrated FP, MNH and nutrition package developed in collaboration with the Ministry of Health (MOH), the Ministry of Gender, Family and Children, and non-governmental organizations in Kinshasa, and on use of a multipurpose smartphone application for counseling. This mobile job aid was developed under the ACQUAL II project, and included modules on pregnancy-, newborn-, and FP-related home visits, a gender inclusion checklist for male partners, and a video module on a wide range of FP and MNH topics to reinforce nursing students’ knowledge of these topics. Nursing students attended a training workshop on the gender-integrated FP/MNH and nutrition package on April 2–16, 2018, and were administered a pre-test exam and a post-test exam.
  To guarantee the quality of home-based service delivery and, upon the request of the *Direction de l’Enseignement des Sciences de Santé* (Department of Health Sciences Education of the MOH, also known as the D6), nursing students were given additional training before the study commenced: A recovery/retraining workshop for those who did not earn 70% in the post-test exam (held on July 20–27, 2018 for 39 nursing students) and refresher training before the start of field work (for 150 nursing students). All students who participated in the recovery/retraining workshop scored between 85% and 98% on the July 2018 post-test exam.
b. Home visits were conducted once a month by third- and fourth-year trained nursing students to provide counseling, services, and referrals for prenatal, postnatal, and newborn care. These visits were scheduled by the D6 to minimize disruptions to the educational program, planned courses, exams, and to student learning in the 11 nursing schools participating in the Momentum pilot. The average number of home visits to FTMs who were ever contacted was 4.8 (SD = 3.2; range 1–21). The number of visits varied depending on the availability of the participant on the scheduled date and time and the extent to which the nursing student deemed additional visits as necessary. Follow-up continued for 16 months. Home visits (and group education sessions discussed below) were suspended in January and February 2019 due to election-related political instability and in May 2019 due to the qualifying exams for 4^th^-year students.
  During the prenatal and postnatal periods, nursing students provided integrated FP, MCH and nutrition education and services during home visits. FP-related health education revolved around birth spacing and the World Health Organization-recommended birth interval of 33 months, partner discussion of FP before delivery, breastfeeding immediately after birth, exclusive breastfeeding, return of the menstrual cycle if the baby is not exclusively breastfed, PPFP methods including the Lactational Amenorrhea Method (LAM) and the intrauterine device, the importance of discussing PPFP with a health worker, side effects of various contraceptive methods, and what to do about side effects. Nursing students also offered a range of contraceptive methods after delivery and during the first 12–14 months postpartum–Implanon NXT, Sayana^®^ Press, progestin-only pills, combined oral contraceptive pills, male condoms, emergency contraception, and Cycle Beads–and method-specific information. If problems were identified, referrals were made to health facilities, specifically high-volume facilities funded by the same donor as Momentum and health facilities utilized by FTMs enrolled in the project and which were assessed as adequate by the MOH. Home-clinic partnerships were established by the project to facilitate referral linkages. Nursing students also dialogued with key household influencers (e.g., parents of the FTM and/or male partner, uncles, aunts, siblings, and others who may have had a positive or negative influence on participants’ FP outcomes and adoption of recommended maternal and child health practices).
  Home visits were supervised by trained instructors/mentors from the nursing students’ respective schools, the Maternal and Newborn Health Coordinator at Tulane International, LLC/Kinshasa, and trained staff from the MOH and the Ministry of Gender, Family, and Children. Nursing students were not paid for home visits but were provided with a transportation allowance and a smart phone used for counseling. All but three of the 11 participating nursing schools were in the intervention health zones. As nursing students were disproportionately female and the project aimed at recruiting an equal number of male and female nursing students, the project had to reach out to nursing schools outside of the intervention health zones. At recruitment, only 24% of nursing students resided in the intervention health zones.
c. Group education sessions were conducted for FTMs once a month and were led by trained nursing students. Group education sessions were based on the DRC-adapted Program M, an approach designed to promote awareness about gender inequities, rights, and health, and improve young women’s agency in interpersonal relationships [24]. Sessions covered the following topics: (1) pregnancy and being a mother, (2) women’s and men’s roles in childcare, (3) sexual and reproductive rights, (4) STI/HIV prevention, (5) FP, (6) gender, (7) empowerment, (8) human rights, and (9) violence (types, cycle, and seeking help). Each session contained activities that encouraged FTMs to question and analyze their own lived experiences, exchange ideas, and contemplate how to make positive changes in their lives and communities. Each session lasted between 45 minutes and two hours and specified the purpose, materials required, recommended time, planning notes, steps for implementing the activities, suggestions of questions to guide discussions about the activity topic, a summary of the key messages, and resource sheets. Starting in December 2018, four Program M sessions were held in each intervention health zone every month (a total of 172 over the life of the project).
d. Group education sessions were also conducted for male partners of FTMs using the DRC-adapted Program P (an approach for engaging men in fatherhood, caregiving, and maternal and child health) [25]. Male partners were identified by the FTMs at enrollment and provided written consent for study participation. For married FTMs, the male partner was presumably the husband, but we did not verify if a reported male partner was the biological father of the newborn. Group education sessions for male partners were led by nursing students and scheduled by the D6. Session topics included (1) father’s expectation, (2) father’s impact/legacy, (3) pregnancy and birth, (4) FP, (5) caregiving, (6) gender, (7) nonviolence, (8) children’s needs and rights, and (9) dimensions of care giving. These sessions aimed to support effective communication between partners about pleasurable and safe sex and shared decision making about sexual and reproductive health, promote the perspective that caregiving of children is the responsibility of both men and women, improve men’s self-confidence and efficacy in caregiving for infants and children, build relationships with men and women that discourage the use of violence as a means to resolve conflict, and encourage couples to teach the values of gender equality to their children and model such equality in their relationships.
  Program P group education sessions provided safe spaces for male partners of FTMs to critically reflect on how social and cultural norms defined fatherhood and men’s and women’s roles, and how gender roles influenced the nature of men’s involvement in their families. Group education sessions were organized once a month in each health zone, following the schedule determined by the D6. Each group education session lasted two to two and a half hours. Each Program P group education session included objectives, suggested time interval for the session, materials required for carrying out the activities, steps for performing the activity or activities included in the session, a summary of key educational messages to be conveyed during and at the close of the session, and supporting information for facilitators.
  Light refreshments were provided for participants. A training-of-trainers workshop on Program M and Program P was conducted from August 6–10, 2018, to improve trainers’ abilities to use tools and tips to engage male partners in MCH, conduct group education sessions, facilitate the different sessions, use materials required for each group activity, and establish teams for training nursing students on Program M and Program P. The group education sessions for both FTMs and their male partners were supervised by trained instructors from the nursing students’ respective schools, the resident Maternal and Newborn Health Coordinator at TILLC/Kinshasa, and trained staff from the MOH and the Ministry of Gender, Family, and Children. Starting in December 2018, four group education sessions were held in each intervention health zone every month (a total of 172 sessions over the life of the project). Group education sessions took place at participating nursing schools and their affiliated health facilities.
e. Social and behavior change communication activities targeted the broader community and key influencers of FTMs. These activities consisted of (a) street theater/short plays conducted in the community; (b) video production among male partners of FTMs on topics related to gender, decision making, and fatherhood; (c) open dialogue among key influencers of FTMs and health workers using a deck of cards with true or false statements about health and social norms; and (d) gatherings of FTMs’ mothers and mothers-in-law, in which positive deviants of a Momentum-targeted health behavior shared their experiences, followed by a question-and-answer session facilitated by a community health agent. Each activity was conducted at least three times per month in each intervention health zone, at locations in or near referral health facilities. Study details can be found in the endline survey report [26].

### Sample size estimation

The sample size was calculated to detect an absolute difference of 10–15 percentage-points in key behavioral indicators with 99% confidence, 99% power, and a 25% cushion for non-response and dropout. At the project proposal stage, it was assumed that the study would have to resort to cluster sampling (assuming a design effect of 2.0) if a cohort follow-up study were not possible due to elections-related political instability. The percentage of babies born to women younger than age 20 who received a postnatal care check within two days of birth, estimated at 6.5% nationwide in the 2013–2014 DRC DHS, was selected as the baseline value for sample size estimation as this indicator had the lowest prevalence among the other 14 behavioral indicators that the project was required to report on. Based on these assumptions, the sample size was estimated at 1,213 FTMs aged 15–24 years and an equal number of male partners in the intervention group and 1,213 FTMs aged 15–24 years and an equal number of male partners in the comparison group at the project design stage [27]. This sample size was powered to detect a 10 percentage points change in postnatal care for the baby within 48 hours of delivery. Although a cohort follow-up study was eventually implemented, and statistical formula suggested a smaller sample of FTMs and male partners, the sample size was not reduced due to the desire to estimate the effects of the intervention separately for adolescent and young FTMs, taking cost into consideration [28]. We also considered the fact that a slightly larger sample size generally increases power.

### Recruitment and attrition

Recruitment of FTMs took place in the community and at 11 designated high-volume maternal health facilities in the intervention and comparison health zones from July 29 to November 23, 2018. Working closely with health zone authorities and community-based health workers, trained recruiters from a collaborating community-based organization went door-to-door in the intervention and comparison health zones to identify eligible FTMs for participation in the Momentum pilot study. Only 40 FTMs were recruited at the health facility level by the study implementing organization, with the assistance of trained prenatal healthcare providers.

Inclusion criteria for FTMs were: (a) age 15–24 years and six-months pregnant with the first child; (b) willing and mentally competent to provide informed consent for the baseline and endline evaluation surveys; (c) able to speak French or Lingala; and (d) usually resides in the intervention or comparison health zones at baseline (i.e., not living in the study area on a temporary basis, for work, vacation or another short-term reason). A total of 2,431 FTMs completed the baseline survey, of whom 1,927 were re-interviewed in the endline survey. The attrition rate was 20.7% overall and was similar in the comparison and intervention HZs (20.0% and 21.4%, respectively, p = 0.394). The main reasons for loss-to follow-up were relocation (the participant travel or moved) and unavailability (the participant was not at home).

In both age groups, the attrition rate was about three to four percentage points higher among ever married FTMs than among those who were never married (see S1 and S2 Tables). Among never married women age 15–19, the attrition rate was identical in the comparison and intervention groups (18.7%). Among their ever-married counterparts, the attrition rate was 22.7% in the comparison group and 23.4% in the intervention group. In the 20–24 age group, the attrition rate was slightly higher in the intervention group than in the comparison group, regardless of marital status (never married 20–24, comparison group = 16.1%; never married 20–24, intervention group = 17.2%; ever married 20–24, comparison group = 19.4%; ever married 20–24 intervention group = 21.6%). As mentioned earlier, the sample size had been inflated by 25% in anticipation of dropout and nonresponse.

Among younger never married FTMs, no significant differences were found in background characteristics between women who were lost to follow-up and those who continued to participate in the study (see S1 Table). In the intervention group, more ever married teenage FTMs who continued participating in the study watched television weekly compared to those lost to follow-up (63% versus 52%; *p* = 0.029; see S1 Table). Among never married FTMs age 20–24, more of those lost to follow-up in the intervention group were Bakongo compared to their retained counterparts (69% versus 31%; p = 0.029) and more of those lost to follow-up in the comparison group had lower than average years of schooling compared to their counterparts who continued to participate in the study (56% versus 29%; p = 0.027) (see S2 Table). Among older FTMs who were ever married, significantly more of those lost to follow-up in the intervention group resided in poor households compared to their counterparts who continued to participate (48% versus 33%; p = 0.028).

### Ethics statement

The study was reviewed and approved by the University of Kinshasa School of Public Health (SPH) Ethics Committee (ESP/CE066/2018) on June 15, 2018, and by the Biomedical Institutional Review Board (IRB) of Tulane University (2018–1028) on July 11, 2018. Authorization to implement the Momentum pilot project was granted by the Secretary General of the Ministère de la Santé Publique (MS.125/SG/PNSR/1358/LBE/2018) on June 11, 2018. Written and electronic consent was obtained by all participants prior to participation. Both IRBs waived consent from a parent or legal guardian for participants younger than age 18 because some of the adolescent FTMs were married and no longer living at home and were considered adults. The authors did not have access to any information that could identify individual participants during and after data collection. All data were de-identified.

### Measures

#### Outcomes

The Integrated Behavior Model (IBM), an extension of the Theory of Planned Behavior, provided a guiding framework for the choice of our contraceptive outcomes (see Montaño and Kasprzyk [29]. The Model asserts that individual behavior is a function of knowledge and skills, attitudes, perceived norms, personal agency, and a set of factors not analyzed in this study (intention, environmental constraints, salience of contraceptive use, and habit (i.e., prior use)). Sociodemographic and environmental characteristics are assumed to operate through model constructs and do not independently contribute to explaining the likelihood of behavioral performance. Prior analysis of the Momentum baseline survey data had shown that these model components (specifically, descriptive norms, perceived community approval of PPFP, normative expectations, PPFP-related self-efficacy and autonomy, and attitude (i.e., rejection of FP myths and misconceptions)) were significant determinants of PPFP intentions [30]. Building on these findings, the present analysis aimed to provide insights into Momentum’s effect on individual constructs of the IBM that were found to be key determinants of PPFP intentions (i.e., attitude, perceived norms, and personal agency) and that were thought to be critical for stimulating positive contraceptive behavior change. Our intention was not to analyze how the Momentum interventions and levels of exposure changed PPFP normative and behavioral outcomes, but to measure the effects of the project’s interventions on IBM components.

Our measures of perceived norms captured normative expectations (beliefs about what other people think one should do), descriptive norms (beliefs about what others do), and injunctive norms (perception of which behaviors others approve or disapprove of) [31], and were measured in both the baseline and endline surveys. Perceived norms were defined as follows for the purpose of the present analysis:

- Normative expectations about partner discussion of PPFP before childbirth: A binary measure indicating strong agreement/agreement with the statement: “Most people who are important to me believe that I ought to discuss use of a method of contraception within the first six weeks following childbirth with my husband/partner before the baby is born.”
- Normative expectations about FP use in the early postpartum period: A binary measure reflecting strong agreement/disagreement with the statement: “Most people who are important to me believe that I ought to start using a method of contraception within the first six weeks following childbirth.”
- Descriptive norms about partner discussion of PPFP before childbirth: A binary measure indicating that the FTMs believed all or more than half of FTMs age 15–24 in her community discussed use of contraceptive methods with their male partner before childbirth.
- Descriptive norms about FP use in the early postpartum period: A binary variable indicating that the FTM believed all or more than half of FTMs 15–24 in her community used contraceptive methods within the first six weeks following childbirth.
- Community injunctive norms about PPFP use: A binary variable indicating that the FTM believed community members would say “good things” (as opposed to saying “bad things” or being indifferent) about women who used a method of contraception within the first six weeks following childbirth.

Personal agency was measured at baseline and endline by PPFP self-efficacy, which was defined as an additive score of responses (on a Likert scale ranging from 1 (not at all confident) to 4 (extremely confident)) to questions measuring the FTM’s level of confidence in overcoming conditions that could constrain FP use in the six-weeks following childbirth. These conditions were: discuss using a method of FP; use FP even if she was afraid that husband/partner would (a) get angry at her, (b) reject her, (c) think she was having sex with someone else, and (d) stop giving her money for food and other necessities; go to a health facility, pharmacy, or store to ask for/buy FP without feeling embarrassed; and refrain from sexual intercourse if she and her husband/partner were getting "turned on" and she could not bring up the subject of protection (baseline α = 0.915; endline α = 0.903). An additive score was constructed to facilitate interpretation and utilization of research results by program managers and policy makers.

There were four contraceptive behavioral outcomes, all of which were measured at endline (given that the FTM was pregnant at baseline).

- Partner discussion of FP in the early postpartum period: binary variable indicating that in the first six weeks following childbirth/pregnancy loss, the FTM discussed use of a method of contraception with her husband/partner.
- Discussion of FP with a health worker in the early postpartum period: binary variable indicating that in the first six weeks following childbirth/pregnancy loss, the FTM discussed FP with a health worker. Although the male partner survey collected data on postpartum family discussion, the present analysis is based on the FTM data set. A comparison of the FTM’s responses with those of her male partner was outside the scope of the present analysis.
- Obtaining a contraceptive method in the early postpartum period: binary variable indicating that, in the first six weeks following childbirth/pregnancy loss, the FTM went to a health facility, pharmacy, or store to get/buy a method of contraception.
- Modern postpartum contraceptive use: binary variable indicating that the FTM started using a modern method of contraception (implant, female and male condom, injectable, intrauterine device, contraceptive pill, Cycle Beads, lactational amenorrhea method, and emergency contraception) 0–11 months after childbirth/pregnancy loss. This analysis was restricted to FTMs who resumed sexual activity within 12 months of childbirth/pregnancy loss. FTMs were asked: “Have you had sexual intercourse since the birth of (NAME OF FIRST CHILD) or since you lost your pregnancy/baby?” Those who responded “yes” to this question were then asked: “For how many months after the birth of (NAME OF FIRST CHILD) or after your pregnancy loss did you not have sexual intercourse?” At the time of the endline interview, 340 FTMs reported that they had not resumed sexual intercourse and an additional 91 reported that they did not know how many months after childbirth/pregnancy loss they resumed sexual intercourse.

#### Intervention exposure

We used several measures of intervention exposure for our bivariate analysis. The total number of Momentum contacts was defined as the number of home visits plus the number of group education sessions (none, 1–3, 4–6, 7–9, and 10+). Level of exposure consisted of three categories: full (participation in both home visits and group education), partial (participation in either home visits or group education), and no exposure (neither). Two variables were specific to FTMs’ participation in home visits. The first variable had four categories and reflected the period(s) in which home visits were received (none; prenatal only; postpartum only; and both prenatal and postpartum). The second variable measured the total number of home visits (none, 1–3, 4–6, and 7+). Two measures were specific to group education sessions: The first reflected participation in group education sessions before and/or after childbirth/pregnancy loss (none, prenatal only, postpartum only, and both prenatal and postpartum). The second measured the total number of group education sessions attended (none, 1–2, 3–4, or 5+).

#### Stratification and control variables

The analysis was stratified by age group (15–19 and 20–24) and marital status at baseline (ever married (defined as those who were formally married or living with a partner in accordance with definitions of marriage used in the DRC DHS), formally engaged (considering the marriage process in the DRC context), widowed, divorced, or separated) versus never married). Age stratification was based on the afore-mentioned five-year age groups to maintain consistency in data reporting, facilitate international comparisons, and allow researchers, health professionals, and policy makers in the DRC to identify and address health issues within standardized age categories used by the government and international organizations for health analysis and programming. All treatment effect models controlled for pre-intervention measures of age (as reported), number of years of schooling (which in the bivariate analysis reflected secondary school completion/higher level versus less education), household wealth (low, medium or high), Bakongo ethnicity (yes versus no), parents’ education (a binary variable reflecting that both parents had attended secondary or higher levels of schooling), weekly television viewing (yes versus no), work in the past 12 months (yes vs. no), gender equity, and power.

Gender equity and power within intimate relationships were measured using the Gender Relations Scale, a 23-item measure assessing a person’s attitude towards gender roles and expectations, decision-making around sex and reproduction, household decision making, violence, and communication [32]. Responses to each item (agreed, disagreed, unsure) were reverse coded as appropriate to indicate a more equitable attitude or greater perceived power, and constituent items summed up to construct the equity subscale (range: 0–16; baseline α = 0.636) and the power subscale (range: 0–7; baseline α = 0.562). A higher score on the equity and power subscales signified more equitable attitudes toward gender roles and more perceived power in the relationship, respectively.

#### Statistical methods

All analyses were performed using Stata software release 17. Within each age and marital status group, differences in sociodemographic characteristics between the comparison and the intervention groups were assessed using Pearson χ^2^ tests. The significance of changes over time in the prevalence of postpartum FP norms were assessed using McNemar’s Test for binary outcomes. The Paired Samples T-test was used for the PPFP self-efficacy score as it was a continuous outcome and normally distributed, with similar spread between the two groups. Significance levels were set at .05 and reported two orders of magnitude below.

To measure the plausible causal effect of Momentum on our outcomes of interest, we first conducted an intent-to-treat analysis, whereby all FTMs were analyzed according to their initial assignment to the intervention group or the comparison group (based on their health zone of residence at baseline), regardless of whether they received any interventions. For outcomes for which FTMs had two observations–one from the baseline survey and one from the endline survey (that is, postpartum FP norms and self-efficacy),–difference-in-differences (DID) models were run. The change in the outcome variable in the intervention group was compared to the change in the outcome in the comparison group, after accounting for FTMs’ baseline characteristics, to give a measure of the average treatment effect (ATE). We fitted random effects probit and linear regression models for the panel data and conducted pairwise comparisons of average marginal effects.

The DID estimation assumes that allocation of the intervention is not determined by the baseline outcome, that there are no spillover effects, and that the intervention and control groups have parallel trends in outcomes. The parallel trend assumption requires that, in the absence of treatment, the difference between the intervention and comparison group is constant over time. A visual diagnostic of this assumption can be obtained by plotting the means of the outcome over time for both groups and is most useful when there are observations over many time points. Momentum data were available for only two time points and more data points would need to be acquired to test the parallel trend assumption. A violation of the parallel trend assumption will lead to biased estimation of the causal effect of Momentum, an issue that will be discussed further in the study limitations section.

For outcomes that were measured only at endline (i.e., our behavioral outcomes), we used treatment effects models with inverse-probability-weighting (IPW). The IPW estimation procedure is based on the following assumptions: the variables that affect both treatment and outcomes are observable; every individual in the study population has a positive probability of receiving either treatment; and independent observations, which ensure that the outcome and treatment for an individual FTM has no effect on the outcome and treatment for any other FTM [33]. We modeled our binary treatment variable, residence in intervention health zones versus comparison health zones at baseline, as a logistic function and our multivalued treatment variables (i.e., level of exposure) as a multinomial logit function. All estimates were adjusted for single years of age, years of schooling, household wealth, ethnicity, parents’ education, weekly television viewing, gender-equitable attitudes, and perceived power in the relationship.

The results of our treatment effects models were expressed as ATEs and 95% confidence intervals (CI). For postpartum contraceptive behaviors, the ATE measured the differences in average outcomes between FTMs in the intervention group and FTMS in the comparison group, after controlling for other factors. A positive ATE meant that the Momentum interventions increased the average predicted outcome while a negative ATE suggested that the Momentum intervention decreased the average predicted outcome. Post-estimation overidentification tests suggested that our covariates were balanced over treatment levels (ever married FTMs: p = 0.92; never married FTMs: p = 0.86). The variance inflation factor had a mean of 1.22, implying that multicollinearity was not of concern.

## Results

### Participants’ characteristics

Table 1 presents the baseline characteristics of the 1,927 FTMs (969 in the comparison group and 968 in the intervention group) who were interviewed in both the baseline and endline surveys. The data were disaggregated by age group, study arm, and marital status. Among both younger and older FTMs, there were some significant differences in background characteristics between the comparison group and the intervention group. The percentage of FTMs who lived in the poorest households and the percentage who reported their ethnic affiliation as Bakongo were higher in the intervention group than in the comparison group. For instance, among teenage FTMs, 35% of those in the comparison group lived in the poorest households compared to 41% of those in the intervention group. Some differences in background characteristics between the comparison group and the intervention group were age-group specific. In the 15–19 age group, a larger share of FTMs in the comparison group were never married (42% versus 36%) but in the 20–24 age group, no significant marital status differences were observed (comparison: 22%; intervention: 24%). Among older FTMs, the comparison group had a significantly larger share of long-term residents (that is, those who had always lived in their community) than the intervention group (33% versus 26%).

**Table 1:** Baseline sociodemographic characteristics of first-time mothers age 15–24, by age group and marital status, Kinshasa.

Baseline Characteristics	Age 15–19		Age 20–24		Total
	Comparison	Intervention	Comparison	Intervention	
% who have always lived in the community					
Ever married	24.9	29.8	26.4	21.9	25.7
Never married	57.0	50.3	56.6	39.8*	51.6
Total	38.4	37.1	32.9	26.2*	33.6
% with complete secondary or higher education					
Ever married	18.3	24.4	65.9	61.9	46.0
Never married	14.0	13.9	61.1	52.2	30.4
Total	16.5	20.7	64.8	59.6	41.3
% residing in the poorest households					
Ever married	33.5	40.3	23.7	34.5***	32.3
Never married	37.1	43.4	31.9	27.4	36.1
Total	35.0	41.4*	25.5	32.8*	33.5
% employed in the past 12 months					
Ever married	28.4	33.7	46.5	44.0	39.3
Never married	23.1	29.5	39.8	31.0	29.7
Total	26.2	32.2*	45.1	40.9	36.4
% with both parents who attained secondary/higher education					
Ever married	80.5	81.9	82.1	76.5	80.3
Never married	74.2	82.1	76.1	78.8	77.9
Total	77.9	82.0	80.8	77.0	79.5
% who watched TV weekly					
Ever married	62.7	63.2	64.4	65.0	63.9
Never married	60.8	52.0	64.6	65.5	59.8
Total	61.9	59.2	64.5	65.1	62.7
% with Bakongo ethnic affiliation					
Ever married	30.7	44.1***	34.9	47.1***	39.5
Never married	23.1	37.0**	29.2	29.2	29.6
Total	27.5	41.6***	33.7	42.8**	36.5
% never married	42.0	35.5*	21.5	24.0	30.4
N					
Ever married	257	315	413	357	1342
Never married	186	173	113	113	585
Total	443	488	526	470	1927

Notes: Data pertain to first-time mothers who were interviewed in both the baseline and endline surveys.

*** p<0.001;

** p<0.01;

* p<0.05.

The significance level pertains to the difference between the comparison group and the intervention group.

Table 1 also shows differences between the comparison group and intervention group within each marital status category. Among both ever married and never married FTMs age 15–19, ethnicity was the only background characteristic that was significantly different between the comparison group and the intervention group, and the difference mirrored the pattern previously presented for the entire age group. In the age group 20–24, the difference between the comparison group and the intervention group in the share of long-term residents was statistically significant only among those who were never married (57% and 40%, respectively). In the same age group, the differences in the share living in poverty and the share who were Bakongo were statistically significant only among the ever married.

Table 1 further shows that there were marked age differences in some background characteristics, regardless of marital status and study arm. Overall, two in five FTMs completed secondary school or had higher levels of education, but two to three times as many FTMs age 20–24 had completed secondary school as those age 15–19. In both the intervention and the comparison group, more teenage FTMs lived in poverty than older FTMs. For instance, among never married FTMs in the intervention group, 43% of 15-19-year-olds lived in the poorest households compared to 27% of 20-24-year-old. Some age differences, such as in employment, were only detected in a specific marital status category. In the total sample, 36% of FTMs were employed in the past 12 months, but among the ever married, older FTMs had higher employment rates than younger FTMs. For instance, the employment rate among ever married FTMs in the intervention group was 34% for those aged 15–19 and 44% for those age 20–24. A similar age pattern in employment was observed among never married FTMs in the comparison group. Weekly exposure to television also varied significantly by age, but only among never married FTMs in the intervention group (age 15–19: 52%; age 20–24: 66%).

Some marital status differences in background characteristics were observed within each age group and the corresponding p-values from chi-square statistics are presented in the text. For example, Table 1 shows that substantially more never married FTMs had always lived in the community compared to those who were ever married. These marital status differences were found in both the comparison and intervention group, irrespective of age. More ever married FTMs had completed secondary/higher education than their never married counterparts, but these educational differences were only statistically significant among 15-19-year-olds in the intervention group (24% versus 14%; p = 0.006) and in the total sample (46% versus 30%; p < 0.001). Marital status variations in employment rates were statistically significant only among women aged 20–24 in the intervention group (ever married: 44%; never married: 31%; p = 0.014). The data also show that almost two in three FTMs watched television weekly. Among FTMs age 15–19 in the intervention group, significantly fewer never married than ever married FTMs watched TV at least once a week (52% versus 63%; p = 0.016). Regarding ethnic affiliation, one in three FTMs in the sample were Bakongo and significantly more ever married than never married FTMs age 20–24 in the intervention group reported their ethnicity as Bakongo.

### Exposure to Momentum interventions

Table 2 presents data on exposure to Momentum interventions among FTMs in the intervention group and permits an assessment of whether the interventions reached the target population and whether ever married and never married adolescent and young FTMs participated equally in the project’s interventions. Four in five FTMs in the intervention group participated in the Momentum interventions and, within each age group, participation rates did not vary significantly by marital status. Only half of FTMs in the intervention group had full exposure to Momentum, and slightly more so among older than younger FTMs. One in three FTMs had partial exposure to Momentum, signifying that they participated in one intervention component (either home visits or group education sessions). On average, FTMs in the intervention group had 5.4 (SD = 4.7) contacts with the Momentum project and, within each age group (and in the overall sample), the mean number of contacts did not vary significantly by marital status (not shown).

**Table 2:** Percent distribution of first-time mothers age 15–24 in the intervention group, by component of the Momentum intervention, age group, and marital status, Kinshasa.

Intervention Component	Age 15–19		Age 20–24		Total
	Ever Married	Never Married	Ever Married	Never Married	
Total number of Momentum contacts					
None	16.5	14.4	14.6	16.8	15.5
1–3	23.2	26.6	21.3	24.8	23.3
4–6	29.2	27.2	29.4	22.1	28.1
7–9	16.5	15.0	19.3	13.3	16.9
10+	14.6	16.8	15.4	23.0	16.3
Level of exposure to Momentum					
None	16.5	14.4	14.6	16.8	15.5
Partial	35.2	38.2	32.2	31.0	34.1
Full	48.3	47.4	53.2	52.2	50.4
Received home visit by Momentum nursing student					
None	19.7	19.1	17.6	20.4	18.9
Prenatal home visit only	20.3	25.4	20.2	16.8	20.8
Postnatal home visit only	5.7	4.6	4.5	2.6	4.7
Both prenatal and postnatal visit	54.3	50.9	57.7	60.2	55.6
Total number of home visits					
None	19.7	19.1	17.6	20.4	18.9
1–3	31.7	37.0	32.2	31.8	32.9
4–6	31.1	27.2	33.9	24.8	30.7
7+	17.5	16.7	16.3	23.0	17.5
Participated in group education sessions					
No	48.6	48.0	43.7	44.2	46.1
Prenatal period only	17.1	13.3	16.3	14.2	15.8
Postnatal period only	20.6	21.4	19.6	26.6	21.1
Both prenatal and postnatal periods	13.7	17.3	20.4	15.0	17.0
Total number of group education sessions					
None	48.6	48.0	43.7	44.3	46.2
1–2	27.0	25.4	27.7	23.9	26.6
3–4	15.6	14.5	16.5	12.4	15.3
5+	6.7	9.2	9.2	13.3	8.9
Do not know	2.1	2.9	2.8	6.2	2.9
N	315	173	357	113	958

Notes: Data pertain to first-time mothers who were interviewed in both the baseline and endline surveys.

Participation rates were higher for home visits than for group education sessions: 80% versus 54%. At least half of FTMs in the intervention group received a home visit in both the prenatal and postnatal periods, with the percentage ranging from 51% among never married women age 15–19 to 60% among never married FTMs age 20–24. Most of the remaining FTMs were visited only in the prenatal period as compared to the postnatal period. Almost half of FTMs received four or more home visits from Momentum nursing students. The average number of home visits from Momentum nursing students was 3.9 (SD = 3.5) and did not vary significantly by marital status.

Fewer than one in five FTMs participated in group education sessions in both the prenatal period and postnatal period. In general, among the remaining FTMs, participation rates were slightly higher in the postnatal period than in the prenatal period. One in four FTMs attended one to two group education sessions and one in five attended three or more sessions. Within each marital status group, participation rates for older and younger FTMs were similar. The average number of group education sessions attended was 1.5 (SD = 1.9), with no statistically significant marital status differences within each age group (not shown). As shown in S3 and S4 Tables, there was negligible crossover between the comparison and intervention groups. Among ever married women in the comparison group, two 15-19-year-olds and five 20-24-year-olds participated in group education sessions.

### Bivariate results

In Table 3, we investigate the prevalence of perceived norms about PPFP before and after the Momentum intervention and the statistical significance of any observed changes. We also examine differences in contraceptive behaviors between the intervention group and the comparison group after the Momentum intervention. In addition, we explore whether the patterns and magnitude of change differed by marital status. The data showed that normative change varied from group to group within the same age bracket and study arm.

**Table 3:** Differences in postpartum family planning normative and behavioral outcomes by survey round, age group and marital status, Kinshasa.

Outcome	Age 15–19						Age 20–24					
	Comparison			Intervention			Comparison			Intervention		
	Baseline	Endline	Sig.	Baseline	Endline	Sig.	Baseline	Endline	Sig	Baseline	Endline	Sig.
% with normative expectations about PPFP discussion with partner												
Ever married	59.1	68.1*		68.5	70.7	ns	67.9	80.0***		68.9	79.0***	
Never married	75.8	74.7	ns	77.9	69.0	ns	68.8	76.9	ns	66.4	79.6*	
% with normative expectations about postpartum FP use												
Ever married	59.9	68.5*		64.4	67.3	ns	68.3	79.0**		70.0	76.8*	
Never married	72.6	78.5	ns	78.8	66.4*		71.7	75.7	ns	72.6	84.1*	
% perceiving descriptive norms about partner PPFP discussion												
Ever married	13.9	19.1	ns	8.9	20.3**		9.9	21.5***		10.1	20.2***	
Never married	15.6	20.4	ns	7.5	20.8**		18.6	23.0	ns	11.5	22.1*	
% perceiving descriptive norms about PPFP use												
Ever married	13.6	14.4	ns	8.9	21.3***		13.3	18.9*		9.5	21.8***	
Never married	16.1	18,3	ns	8.7	20.8**		17.7	23.0	ns	12.4	22.1	ns
% perceiving community will say good things about PPFP users												
Ever married	35.0	26.8*		25.4	31.4	ns	40.2	24.4***		25.5	27.7	ns
Never married	43.0	33.9*		25.4	29.5	ns	40.7	35.4	ns	27.4	32.7	ns
Mean PPFP personal agency score												
Ever married	18.1	18.5	ns	19.3	19.6	ns	18.7	19.1	ns	19.4	19.8	ns
Never married	18.9	18.7	ns	18.7	18.6	ns	19.7	18.5**		19.5	19.4	ns
% discussing FP in early postpartum period with male partner												
Ever married		40.9			61.6***			38.2			50.3*	
Never married		38.2			58.3***			47.0			70.0***	
% discussing FP in early postpartum period with health worker												
Ever married		42.8			69.5***			44.6			71.7***	
Never married		46.2			60.1**			38.9			67.3***	
% obtaining contraceptives in the early postpartum period												
Ever married		14.8			32.7***			19.9			29.4**	
Never married		16.1			22.5	ns		19.5			39.8***	
% using PPFP (a)												
Ever married		52.3			63.6*			43.5			55.7**	
Never married		39.5			64.0***			45.9			55.0	ns
N												
Ever married	257			315			413			357		
Never married	186			173			113			113		

Note: n.a.–Not applicable. Data were collected only at endline; FP–family planning; PPFP—postpartum family planning.

^(a)^ Restricted to FTMs who resumed sexual activity 0–11 months after childbirth/pregnancy loss.

*** p<0.001;

** p<0.01;

* p<0.05;

ns-Not statistically significant. The significance level pertains to the difference between the baseline survey and the endline survey for outcomes measured in both surveys and to the difference between the intervention group and the comparison group for outcomes that were only measured in the endline survey.

For use of PPFP, the Ns for the comparison group were as follows: ever married age 15–19 = 199; never married age 15–19 = 129; ever married age 20–24 = 356; never married age 20–24 = 85. The corresponding Ns for the intervention group were as follows: ever married age 15–19 = 242; never married age 15–19 = 100; ever married age 20–24 = 305; never married age 20–24 = 80.

In the 15–19 age group, normative expectations about partner discussion of FP before childbirth changed significantly only among ever married FTMs in the comparison group (from 59% to 68%). Although there were no significant changes in these normative expectations among never married FTMs age 15–19 in the intervention group, among their older counterparts, the percentage reporting that most people who were important to them expected them to discuss PPFP use with their husband/partner before childbirth increased significantly between the baseline and the endline surveys, from 66% to 80%. Normative expectations around use of FP in the early postpartum period showed similar patterns, the only difference being the significant unexpected decline among never married women age 15–19 in the intervention group (79% at baseline vs 66% at endline).

Table 3 also shows changes in descriptive norms around (i.e., the perceived prevalence of) partner discussion of postpartum FP before the baby’s birth. The percentage of FTMs who believed that most new mothers in the community discussed using a method of contraception within the first six following childbirth with their husband/partner before the baby’s birth increased significantly between the baseline and endline surveys among ever married women, except those age 15–19 in the comparison group. No significant change occurred among never married FTMs, with one exception: never married 15-19-year-olds in the intervention group, among whom the perceived prevalence of partner discussion increased from 9% at baseline to 21% at endline. Descriptive norms around PPFP use in the early postpartum period showed a similar pattern.

In the comparison group, there was a decrease in the percentage of FTMs reporting that the community would say good things about women who used FP in the early postpartum period. This decrease occurred among both younger and older ever married FTMs, and among never married teenagers. FTMs in the intervention group reported similar levels of perceived community support for FP use in the early postpartum period at baseline and endline, regardless of marital status and age. No significant differences in pre-intervention normative outcomes were detected between FTMs who were lost to follow-up and those who continued to participate in Momentum (see S5 and S6 Tables).

Regarding the behavioral outcomes, the bivariate analyses showed that in each age group, FTMs in the intervention group reported a higher prevalence of discussion of FP with their male partner and a health worker in the early postpartum period, of obtaining contraceptives in the same period, and of using a modern contraceptive within 12 months of childbirth/pregnancy loss than FTMs in the comparison group. These findings were observed for both ever married and never married FTMs. There were two exceptions. We did not find significant differences between the comparison group and the intervention group in the percentage of never married FTMs age 15–19 who reported obtaining contraceptives in the early postpartum period and the percentage of never married FTMs age 20–24 who reported using modern contraceptives 0–11 months after childbirth/pregnancy loss.

We also observed some significant marital status differences in the prevalence of contraceptive behaviors in the intervention group. For example, among FTMs age 20–24 in the intervention group, the never married had a higher prevalence than the ever married of partner discussion of FP in the early postpartum period (70% versus 50%) and of obtaining contraceptives in the early postpartum period (40% versus 29%), but not of using modern contraception 0–11 months after childbirth/pregnancy loss. In the intervention group, both ever married and never married adolescent FTMs had higher rates of postpartum modern contraceptive use than their older counterparts.

### Treatment effects

Table 4 presents the results of an intent-to-treat analysis. All participants were included in the statistical analysis and analyzed according to their original group assignment, regardless of whether they received the Momentum interventions. We assessed the effectiveness of the Momentum interventions on PPFP-related norms and behaviors, after controlling for observed baseline characteristics that could influence participation in the project. For the normative outcomes and personal agency, the ATEs are difference-in-differences estimates that compare the changes over time between the intervention and comparison groups. For the behavioral outcomes, the ATEs are single difference estimates that compare the outcomes in the intervention group with the outcomes in the comparison group at a single point in time following the Momentum intervention (i.e., four to seven months after project implementation) using treatment effects models. We compared intervention effects between four groups of FTMs: adolescents (age 15–19) and young women (age 20–24), separately ever married and never married.

**Table 4:** Average treatment effects for family planning normative and behavioral outcomes by age group and marital status (intent-to-treat analysis), first-time mothers age 15–24, Kinshasa.

Outcome	Age 15–19						Age 20–24					
	Ever Married			Never Married			Ever Married			Never Married		
	ATE	p-value	95% CI	ATE	p-value	95% CI	ATE	p-value	95% CI	ATE	p-value	95% CI
Normative expectations about partner discussion of PPFP	0.034	0.489	(-0.063, 0.132)	0.092	0.123	(-0.025, 0.210)	0.079	0.062	(-0.004, 0.163)	0.220	0.003	(0.075, 0.366)
Normative expectations about PPFP use	0.026	0.603	(-0.073, 0.126)	-0.017	0.774	(-0.130, 0.097)	0.038	0.375	(-0.046, 0.122)	0.240	0.002	(0.091, 0.388)
Descriptive norms about partner discussion of PPFP	0.051	0.215	(-0.030, 0.132)	0.079	0.122	(-0.021, 0.180)	-0.015	0.652	(-0.080, 0.050)	0.063	0.384	(-0.078, 0.204)
Descriptive norms about PPFP use	0.110	0.005	(0.033, 0.187)	0.095	0.075	(-0.010, 0.200)	0.067	0.049	(0.000, 0.134)	0.045	0.516	(-0.091, 0.182)
Perceived community members will say good things about PPFP users	0.145	0.006	(0.042, 0.247)	0.127	0.035	(0.009, 0.245)	0.180	<0.000	(0.093, 0.267)	0.107	0.173	(-0.047, 0.262)
PPFP personal agency score	0.534	0.030	(0.053, 1.014)	0.097	0.762	(-0.528, 0.721)	0.839	<0.001	(0.429, 1.249)	0.805	0.026	(0.095, 1.515)
In early postpartum period, discussed FP with male partner	0.179	<0.001	(0.098, 0.261)	0.131	0.011	(0.029, 0.232)	0.233	<0.001	(0.164, 0.302)	0.241	<0.001	(0.121, 0.362)
In early postpartum period, discussed FP with health worker	0.260	<0.001	(0.179, 0.341)	0.128	0.015	(0.025, 0.231)	0.275	<0.001	(0.208, 0.343)	0.306	<0.001	(0.185, 0.427)
In early postpartum period, obtained a contraceptive method	0.190	<0.001	(0.123, 0.257)	0.061	0.139	(-0.020, 0.143)	0.097	0.002	(0.035, 0.158)	0.220	<0.001	(0.104, 0.336)
PPFP use (a)	0.114	0.017	(0.020, 0.208)	0.251	<0.001	(0.122, 0.380)	0.113	0.004	(0.036, 0.190)	0.098	0.193	(-0.050, 0.245)
N	570			358			770			226		

Notes: Regressions control for the following baseline characteristics of the FTM: single years of age, years of schooling, household wealth, ethnicity, parental education, weekly television exposure, gender equity score, and power score.

(b) Restricted to FTMs who resumed sexual activity 0–11 months after childbirth/pregnancy loss.

FP–family planning, PPFP–postpartum family planning.

Our main findings were that Momentum interventions had stronger effects on postpartum FP behaviors than on postpartum FP norms (i.e., more positive behavioral effects than normative effects) and no effect on normative change among never married 15-19-year-olds. Among never married FTMs age 20–24, there was a 22 percentage points increase in the probability of the FTM perceiving that significant others expected her to discuss postpartum FP with her male partner before childbirth in the intervention group compared with the comparison group. There was also an increase of 24 percentage points in the probability that a never married 20-24-year-old FTM perceived significant others to expect her to use FP in the early postpartum period in the intervention group compared with the comparison group. No intervention effects were detected on (a) normative expectations about postpartum FP discussion and use among FTMs age 15–19 (regardless of marital status) and among ever married FTMs age 20–24; and (b) descriptive norms about postpartum FP discussion. However, Momentum had a small positive effect on descriptive norms about postpartum FP use among ever-married FTMs (age 15–19: ATE = 0.110, 95% CI = 0.033, 0.187; age 20–24: ATE = 0.067, 95% CI = 0.000, 0.134).

Concerning the probability of perceiving that the community would say “good things” about women who used FP in the early postpartum period, there was a positive and statistically significant intervention effect in all subgroups except never married 20-24-year-olds. Additionally, among ever married FTMs, the postpartum FP personal agency scores were significantly higher in the intervention group than in the comparison group, regardless of age. Similar results were obtained among never married FTMs age 20–24. Momentum had no effect on postpartum FP personal agency among never married FTMs age 15–19.

Some of the largest intervention effects on contraceptive behaviors were seen for discussion of FP with the male partner and a health worker in the early postpartum period, with the intervention group exhibiting significantly greater outcomes than the comparison group, after adjusting for covariates. The ATEs were larger among older than younger FTMs. For discussion of FP with a health worker after childbirth/pregnancy loss, ATEs ranged from 13 percentage points (ATE = 0.128, 95% CI = 0.05, 0.231) among never married FTMs age 15–19 to 31 percentage points (ATE = 0.306, 95% CI = 0.185, 0.427) among never married FTMs age 20–24. In terms of obtaining a method of contraception in the early postpartum period, the intervention worked for ever married 15-19-year-olds (ATE = 0.190, 95% CI = 0.123, 0.257), but not for 15-19-year-olds who were never married. In the 20–24 age group, intervention effects on this outcome were statistically significant among both ever married and never married FTMs. Although Momentum had a significant positive effect (ranging from 10 to 25 percentage points) on use of a modern contraceptive method within the first 12 months of childbirth/pregnancy loss, this effect was not significant for FTMs age 20–24 who were never married.

We conducted sensitivity analyses to assess the robustness of our results by estimating the ATE for each behavioral outcome using: (a) inverse probability weighting (IPW); (b) augmented inverse probability weighting (AIPW); and (c) propensity score matching (PSM). The AIPW estimator requires us to use a model to predict the outcomes for each FTM and another model to predict the treatment status (i.e., intervention group or comparison group). This modelling approach is more robust to misspecification but does not perform well when the predicted treatment probabilities are too close to zero or one. PSM estimates the average treatment effect by matching FTMs that have similar probabilities of receiving the intervention. The propensity score is the probability that FTMs with certain characteristics will be assigned to the intervention group. A description of the methods, assumptions, and statistical formulae for these treatment effects estimators is beyond the scope of the present paper; details can be found in the Stata Version 17 Treatment Effects Reference Manual [33].

S7 Table presents the results of our sensitivity analysis. Each treatment effects model was run separately for ever married FTMs age 15–19, never married FTMs age 15–19, ever married FTMs age 20–24, and never married FTMs age 20–24 (a total of 48 models). For all outcomes analyzed, IPW and AIPW produced estimates that were close; the differences in the estimated probabilities of the behavioral outcomes ranged from zero to 0.002 for partner discussion of FP in the early postpartum period; from zero to 0.008 for discussion of FP with a health worker in the early postpartum period; from 0.001 to 0.005 for obtaining a contraceptive method in the early postpartum period; and from 0.001 to 0.01 for modern postpartum contraceptive use. The differences in the ATEs produced by the IPW and PSM were slightly larger. These differences ranged from 0.013 to 0.061 for partner discussion of FP in the early postpartum period; from 0.003 to 0.007 for discussion of FP with a health worker in the early postpartum period; from 0.008 to 0.024 for obtaining a contraceptive method in the early postpartum period; and from 0.003 to 0.101 for modern postpartum contraceptive use. We concluded, therefore, that our estimates of the effect of Momentum were robust except in three instances in which the level of significance of the project’s effect on selected behavioral outcomes declined to ten percent: (a) partner discussion of FP among never married 15-19-year-old FTMs (IPW: p = 0.011; PSM: p = 0.095), (b) modern postpartum contraceptive use among ever married 15-19-year-olds (IPW: p = 0.017; PSM: p = 0.058), and (c) modern postpartum contraceptive use among ever married 20-24-year-olds (IPW: p = 0.004; PSM: p = 0.074).

Results on the effect of the level of exposure to Momentum interventions on outcomes of interest are shown in Table 5. These ATEs were estimated using IPW estimation procedures and are important for informing program decision-making processes. For all behavioral outcomes among ever married FTMs age 15–19, the ATE for partial exposure relative to no exposure was similar in magnitude to the ATE for full exposure relative to no exposure, the only exception being PPFP use. Regardless of marital status and age, the ATE for partial exposure relative to no exposure to Momentum was not statistically significant for postpartum FP use. The main difference between ever married and never married FTMs was that partial relative to no exposure to Momentum had statistically significant effects on FP discussion with the male partner and health worker, and on obtaining a contraceptive method in the early postpartum period among ever married FTMs, but not among their never married counterparts. Similar marital status differences in the ATE for partial exposure were observed for discussion of FP with the male partner and a health worker among FTMs age 20–24.

**Table 5:** Effect of the level of exposure to Momentum interventions on family planning normative and behavioral outcomes, by marital status and age group, first-time mothers age 15–24, Kinshasa.

Outcome	Age 15–19						Age 20–24					
	Ever Married			Never Married			Ever Married			Never Married		
	ATE	p-value	95% CI	ATE	p-value	95% CI	ATE	p-value	95% CI	ATE	p-value	95% CI
Normative expectations: partner discussion of PPFP												
Partial vs. no exposure	0.032	0.621	(-0.095, 0.159)	0.099	0.239	(-0.065, 0.263)	0.007	0.906	(-0.111, 0.126)	0.146	0.174	(-0.064, 0.356)
Full vs. no exposure	0.062	0.271	(-0.049, 0.173)	0.028	0.695	(-0.112, 0.168)	0.129	0.009	(0.032, 0.225)	0.232	0.006	(0.066, 0.397)
Normative expectations: PPFP use												
Partial vs. no exposure	0.008	0.903	(-0.123, 0.139)	-0.037	0.651	(-0.195, 0.122)	-0.037	0.531	(-0.157, 0.081)	0.291	0.007	(0.079, 0.503)
Full vs. no exposure	0.059	0.309	(-0.054, 0.172)	0.020	0.772	(-0.116, 0.157)	0.129	0.009	(0.032, 0.226)	0.160	0.059	(-0.006, 0.327)
Descriptive norms: partner discussion of PPFP												
Partial vs. no exposure	0.055	0.293	(-0.048, 0.158)	0.029	0.640	(-0.093, 0.152)	-0.032	0.461	(-0.118, 0.053)	0.091	0.274	(-0.072, 0.253)
Full vs. no exposure	-0.003	0.948	(-0.096, 0.090)	0.150	0.022	(0.022, 0.279)	-0.003	0.934	(-0.084, 0.077)	0.037	0.685	(-0.140, 0.214)
Descriptive norms: PPFP use												
Partial vs. no exposure	0.124	0.016	(0.023, 0.226)	0.000	0.994	(-0.122, 0.123)	0.047	0.313	(-0.044, 0.137)	0.073	0.365	(-0.086, 0.232)
Full vs. no exposure	0.058	0.216	(-0.034, 0.149)	0.150	0.029	(0.015, 0.285)	0.048	0.245	(-0.033, 0.130)	-0.013	0.879	(-0.186, 0.160)
Perceived community members would say “good things” about PPFP users												
Partial vs. no exposure	0.179	0.009	(0.045, 0.311)	0.047	0.550	(-0.106, 0.201)	0.085	0.170	(-0.037, 0.207)	0.170	0.111	(-0.039, 0.379)
Full vs. no exposure	0.125	0.040	(0.006, 0.245)	0.099	0.174	(-0.044, 0.241)	0.194	<0.001	(0.092, 0.296)	0.013	0.886	(-0.170, 0.196)
Personal agency score												
Partial vs. no exposure	0.620	0.053	(-0.008, 1.248)	0.650	0.128	(-0.186, 1.486)	0.048	0.871	(-0.531, 0.627)	1.060	0.041	(0.042, 2.079)
Full vs. no exposure	0.627	0.031	(0.059, 1.198)	0.058	0.881	(-0.709, 0.826)	1.045	<0.001	(0.556, 1.534)	0.396	0.396	(-0.443, 1.235)
In the early postpartum period, discussed FP with male partner												
Partial vs. no exposure	0.184	0.001	(0.079, 0.289)	0.045	0.540	(-0.098, 0.187)	0.187	<0.001	(0.088, 0.286)	0.072	0.425	(-0.105, 0.249)
Full vs. no exposure	0.192	<0.001	(0.096, 0.288)	0.213	0.001	(0.087, 0.338)	0.311	<0.001	(0.235, 0.386)	0.226	<0.001	(0.083, 0.368)
In the early postpartum period, discussed FP with health worker												
Partial vs. no exposure	0.231	<0.001	(0.130, 0.333)	-0.017	0.810	(-0.154, 0.120)	0.176	<0.001	(0.077, 0.276)	0.095	0.276	(-0.075, 0.265)
Full vs. no exposure	0.288	<0.001	(0.198, 0.379)	0.222	<0.001	(0.100, 0.344)	0.335	<0.001	(0.261, 0.410)	0.326	<0.001	(0.193, 0.459)
In early postpartum period, obtained a contraceptive method												
Partial vs. no exposure	0.193	<0.001	(0.095, 0.291)	-0.041	0.379	(-0.133, 0.051)	0.065	0.152	(-0.024, 0.154)	-0.025	0.740	(-0.174, 0.124)
Full vs. no exposure	0.180	<0.001	(0.094, 0.267)	0.087	0.123	(-0.023, 0.197)	0.010	0.010	(0.023, 0.176)	0.242	0.001	(0.102, 0.382)
PPFP use (a)												
Partial vs. no exposure	0.052	0.410	(-0.072, 0.176)	0.140	0.114	(-0.034, 0.313)	-0.019	0.740	(-0.128, 0.091)	-0.073	0.502	(-0.286, 0.140)
Full vs. no exposure	0.222	<0.001	(0.118, 0.325)	0.383	<0.001	(0.251, 0.515)	0.191	<0.001	(0.101, 0.281)	0.289	0.001	(0.124, 0.454)
**N**												
No exposure	307			211			459			132		
Partial exposure	113			65			121			35		
Full exposure	150			82			190			29		
Total	570			358			770			226		

Notes: Regressions control for the following baseline characteristics of the FTM: single years of age, years of schooling, household wealth, ethnicity, parental education, weekly television exposure, gender equity score, and power score; FP–family planning, PPFP–postpartum family planning.

^(a)^ Restricted to FTMs who resumed sexual activity 0–11 months after childbirth/pregnancy loss.

As Table 5 shows, in the 20–24 age group, full exposure was consistently characterized by larger increases in average behavioral outcomes than partial exposure, after adjusting for covariates. These results were observed for normative expectations, perceived community support for use of FP in the early postpartum period, and use of a modern contraceptive within 12 months of childbirth/pregnancy loss. For example, among never married women age 20–24, the estimated ATE of going from no exposure to partial exposure was -0.073 (95% CI = (-0.286, 0.140) and the estimated ATE of going from no exposure to full exposure was 0.289 (95% CI = (0.124, 0.454) for postpartum FP use, after adjusting for covariates.

It is worth noting that for perceived community support for FP use in the early postpartum period and personal agency, the effects of full exposure relative to no exposure were statistically significant among ever married FTMs in both age groups, but not among their counterparts who were never married.

We used reverse adjacent contrasts to determine whether there was a significant change in our normative and behavioral outcomes if the FTM was fully exposed as opposed to partially exposed to Momentum interventions. As Table 6 shows, the gains to participation in both Momentum interventions relative to one intervention were not statistically significant for normative outcomes, except normative expectations about FP use in the early postpartum period among FTMs age 20–24, irrespective of marital status. In both age groups, the ATEs associated with full versus partial exposure were larger among never married compared to ever married FTMs for all behavioral outcomes. On average, participation in both home visits and group education sessions increased an FTM’s probability of FP use within 12 months of -childbirth/pregnancy loss by 17–36 percentage points relative to participation in only one of these interventions. Having full versus partial exposure to Momentum did not have a significant effect on nine out of 10 outcomes among ever married 15-19-year-old FTMs. Concerning personal agency, ever married FTMs age 20–24 were the only subgroup for which full vs partial exposure made a significant difference (ATE = 0.997, 95% CI = 0.338, 1.656; p = 0.003).

**Table 6:** Effects of full exposure versus partial exposure to Momentum interventions on family planning normative and behavioral outcomes, by marital status and age group, first-time mothers age 15–24, Kinshasa.

Outcome	Age 15–19						Age 20–24					
	Ever Married			Never Married			Ever Married			Never Married		
	ATE	p-value	95% CI	ATE	p-value	95% CI	ATE	p-value	95% CI	ATE	p-value	95% CI
Normative expectations: partner discussion of PPFP	0.030	0.669	(-0.108, 0.169)	-0.071	0.456	(-0.257, 0.115)	0.121	0.069	(-0.010, 0.252)	0.086	0.255	(-1.807, 0.479)
Normative expectations: PPFP use	0.050	0.488	(-0.092, 0.193)	0.057	0.540	(-0.124, 0.238)	0.167	0.013	(0.036, 0.298)	0.226	<0.001	(0.083, 0.368)
Descriptive norms: partner discussion of PPFP	-0.058	0.321	(-0.174, 0.057)	0.121	0.115	(-0.030, 0.272)	0.029	0.578	(-0.073, 0.130)	-0.054	0.597	(-0.254, 0.146)
Descriptive norms: PPFP use	-0.067	0.257	(-0.182, 0.049)	0.149	0.055	(-0.003, 0.302)	0.002	0.975	(-0.103, 0.107)	-0.087	0.390	(-0.285, 0.111)
Perceived community members would say good things about PPFP users	-0.052	0.489	(-0.201, 0.096)	0.052	0.565	(-0.124, 0.228)	0.108	0.120	(-0.028, 0.245)	-0.156	0.195	(-0.393, 0.080)
Personal agency score	0.008	0.983	(-0.703, 0.719)	-0.591	0.237	(-1.571, 0.388)	0.997	0.003	(0.338, 1.656)	-0.664	0.255	(-1.807, 0.479)
In the early postpartum period, discussed FP with male partner	0.008	0.899	(-0.111, 0.127)	0.168	0.046	(0.003, 0.333)	0.124	0.023	(0.017, 0.230)	0.154	0.128	(-0.044, 0.352)
In the early postpartum period, discussed FP with health worker	0.057	0.315	(-0.054, 0.167)	0.239	0.003	(0.084, 0.394)	0.159	0.003	(0.053, 0.265)	0.231	0.014	(0.048, 0.415)
In early postpartum period, obtained a contraceptive method	-0.013	0.831	(-0.130, 0.105)	0.128	0.040	(0.006, 0.250)	0.035	0.521	(-0.071, 0.140)	0.267	0.004	(0.088, 0.446)
PPFP use (a)	0.170	0.012	(0.037, 0.302)	0.244	0.009	(0.060, 0.428)	0.210	0.001	(0.086, 0.333)	0.362	0.002	(0.128, 0.596)
N												
No exposure	307			211			459			132		
Partial exposure	113			65			121			35		
Full exposure	150			82			190			59		

Notes: Regressions control for the following baseline characteristics of the FTM: age, years of schooling, household wealth, ethnicity, parental education, weekly television exposure, gender equity score, and power score.

^(a)^ Restricted to FTMs who resumed sexual activity 0–11 months after childbirth/pregnancy loss.

FP–family planning, PPFP–postpartum family planning.

## Discussion

This study evaluated whether integrated FP/MCH and nutrition home visits and group education sessions conducted by trained nursing students had positive effects on postpartum FP-related norms and behaviors among first-time mothers age 15–24 in Kinshasa. We found robust evidence linking the Momentum interventions to increased discussion of postpartum FP with the male partner and a health worker in the early postpartum period, and an increased probability of obtaining a contraceptive method in the early postpartum period and using a modern method within 12 months of childbirth/pregnancy loss. Momentum’s effects of contraceptive behaviors did not appear to vary much by marital status and were generally larger for older than younger FTMs, one exception being PPFP use among never married FTMs. Overall, full exposure to Momentum had consistently larger effects on postpartum FP behaviors than partial exposure and, among teenage FTMs, the never married appeared to benefit more from full versus partial exposure than the ever married.

Our findings are consistent with previous studies focusing on community-based interventions implemented by community health workers. Although these studies did not examine the difference in the impact of their interventions by age and marital status groups, they found that engaging adolescents and youth through community-based programs such as home counseling and education positively impacted contraceptive behaviors [34–38]. Evidence also suggests that integration of FP with other programs and repeated antenatal and postnatal interventions can be effective in improving PPFP outcomes [39]. Other studies have also found that interventions with multiple strategies and higher dosage are more likely to be effective in changing behaviors than single and lower dosage interventions [40,41].

In our study, two potential mechanisms may have contributed to significantly improved postpartum contraceptive behaviors across age and marital status groups. Home visits reduced geographic barriers and opportunity costs to FP care seeking (e.g., time, cost of contraceptives, distance, transport costs) by bringing free services into the home. This probably helped more FTMs access their desired FP methods. Nursing students also helped to bridge gaps in FP-related care for new mothers by building trust, improving access, and promoting FP. In addition, nursing students helped young mothers, their male partners, and key household influencers understand the importance of improved birth spacing for family welfare, facilitated informed method choice, supported FTMs to overcome barriers to contraceptive use and, through referrals, served as a bridge between the community and the health facility.

Momentum had weaker and mixed effects on postpartum FP-related norms and personal agency. Our results pointed to the heterogeneity of FTMs and highlighted the intersectionality between young maternal age and single motherhood. Momentum had no effect on (a) normative expectations among both ever married and never married 15-19-year-olds; (b) descriptive norms among never married 15-19-year-olds; (c) the perception among never married mothers age 20–24 that community members would say “good things: about postpartum FP users; and (d) postpartum FP agency among never married teenage mothers. Overall, we saw fewer program effects on never married 15-19-year-olds compared to never married 20–24-year-olds (four versus seven of 10 outcomes), highlighting the increased vulnerability of these adolescent mothers as opposed to slightly older ones.

Given the lack of significant age and marital status differences in exposure to the project’s interventions, it is possible that subgroup differences in Momentum’s effects on normative outcomes may have been driven by differences in family and community attitudes toward unmarried teenage mothers. Despite the project’s efforts to create an enabling environment for broad-based gender and community norm change, there may have still been negative stereotypes surrounding unmarried teenage mothers. Qualitative research among adolescents and young adults in the DRC has revealed that attitudes toward premarital sex are generally favorable among peers, but not among adults [42]. This finding received indirect support in the Momentum endline survey, which showed that the percentage of FTMs who reported their mother/mother figure or father/father figure was “very unhappy” with the pregnancy was at least four times as high as for the husband/partner or his mother/mother figure. More adolescent than young adult FTMs perceived their mother to be “very unhappy” with the pregnancy [26]. Unfortunately, the Momentum endline survey report did not disaggregate parental unhappiness with the pregnancy by the FTM’s marital status.

This study has several limitations. The sample was not representative of FTMs in Kinshasa, which limited the generalizability of the study’s findings to the broader population. Selection bias is an important concern. The unrepresentative sample may have also led to inflation or underestimation of the true effect size, misleading conclusions about the importance of an effect, and difficulties in obtaining consistent results if the study is replicated. Although we conducted sensitivity analyses to assess the robustness of our estimated intervention effects and checked for covariate balance in our total effects models, it should be considered that violations of the assumptions underlying difference-in-difference models and total effects models with inverse probability weighting could lead to biased and inefficient estimates.

As the outcomes were reliant on self-reports, they were subject to social desirability bias. Measures of social norms around partner discussion of FP and its use were limited to the early postpartum period, constraining our understanding of the effect of the project on FP norms in the extended postpartum period. It should also be noted that these norms may have been misperceived. We did not interview persons named as key influencers of contraceptive use, which would have enabled us to better understand normative change, or the lack thereof. While the intervention consisted of both home visits and group education sessions, the evaluation was not designed to assess the effects of these components separately. In situations where the ATEs for partial exposure (relative to no exposure) were significant, we could not pinpoint which intervention (home visits or group education) had a larger effect on observed normative shifts or postpartum contraceptive behaviors.

Almost half of the participants did not participate in group education sessions, indicating low reach for this intervention component. Also, there may have been unobserved variables (e.g., motivation or neighborhood characteristics) that determined FTMs’ recall of program exposure. For example, if recall of program exposure was higher among more motivated FTMs, this could have led to a spurious positive effect of the level of program exposure on contraceptive outcomes. A fifth of the sample was lost to follow-up, but sample size estimates had been inflated by 25% to account for this possibility. As we did not assign FTMs randomly to an intervention group and a control group, we could not conclusively prove that the Momentum project caused observed normative shifts or increased postpartum family planning self-efficacy or behavior. Nevertheless, our results strongly suggested that Momentum’s integrated home visits and group education sessions were associated with gains in postpartum contraceptive behaviors in each marital status and age group, in postpartum FP agency among ever married FTMs regardless of age, and in normative expectations about partner discussion of FP before childbirth and about FP use in the early postpartum period among never married FTMs age 20–24.

Our research contributes to the growing evidence on strategies for increasing PPFP use among adolescent girls age 15–19 and young women age 20–24 in sub-Saharan Africa. The present study has demonstrated that offering a community-based integrated package of counseling, services, and referrals for FP, MNH and nutrition has the potential to increase partner and health worker discussion about and use of postpartum FP by both unmarried and married FTMs. Our finding of fewer statistically significant project effects among teenage never married mothers highlights their greater vulnerability.

Although our study has provided some evidence of programmatic effectiveness, cost and the potential for sustainability are important considerations when making strategic choices for the successful institutionalization of evidence-based interventions. A cost analysis of the Momentum intervention had indicated that second to staff salaries, training activities aimed at achieving higher learning goals were the costliest items and required considerable resource mobilization and logistics to ensure an optimal learning environment. During the Momentum pilot, training costs were driven by rentals, food, transportation reimbursement for the nursing students and the trainers, and per diems for the trainers. Training costs were higher for home visits than for group education sessions and were driven by the challenges the study faced keeping FTMs in the cohort, particularly the unmarried, due to their high mobility during and after pregnancy. During the pilot study, the mobility of unmarried FTMs around childbirth prompted two campaigns to track “lost” FTMs across Kinshasa. The FTMs retrieved through these campaigns were seen in their new location for the remaining time. Home visits delivered to FTMs located outside Momentum’s catchment areas increased transportation and supervision costs, especially during the study’s final months [43].

Drawing on cost considerations and lessons learned from the implementation of the Momentum pilot project, a study on the acceptability and feasibility of using medical and nursing students to instruct clients in DMPA-SC self-injection, and the successful integration of contraceptive service delivery into nursing school training [23,44], the DRC Government’s “Momentum Approach Implementation Plan,” which was validated by the Technical Coordination Committee of the MOH in April 2022, includes strategic adjustments and adaptations to increase the sustainability of the Momentum approach. The plan entails: (1) introducing gender-integrated community-based maternal, newborn and child health (MNCH) and family planning (FP) service delivery in nursing school training: both classroom instruction plus a two-year field practicum in which students offer MNCH/FP services through home visits and clinic-based group education sessions; (2) considering all nulliparous pregnant women, regardless of age and gestational stage during home visits; (3) adopting a non-cohort-based approach; (4) creating nursing student pairs for the field-base internship without considering sex (due to the relative lack of male students); (4) following up children for 24 months; and (5) implementing the community-based internship in the catchment area of the nursing student’s school. At the time of writing, the nursing curriculum had been revised and was being piloted in selected nursing schools in Kinshasa. A process evaluation was underway to identify barriers to and facilitators of the successful institutionalization of the Momentum approach, help ensure the quality of service provision, and provide insights for further adaptation as the Momentum-integrated nursing curriculum is expanded.

## Conclusions

Our results suggested that a “one-size-fits-all” approach may not be optimal for improving postpartum FP-related norms and personal agency among FTMs age 15–24, and that interventions need to be tailored for different age and marital status groups and social contexts. Program efforts to increase personal agency around postpartum FP use need to be intensified among never married 15-19-year-old FTMs. While increased access to contraceptives is essential, fostering normative change in support of PPFP uptake is important for future programming. As perceived PPFP norms may shape FTMs’ attitudes, beliefs, and behaviors if those norms are internalized, public dialogue will be required to correct any misperceptions about what others in their community do and approve of, especially among never married 15-19-year-old FTMs.

## Supporting information

S1 Table Percent distribution and mean age of first-time mothers age 15–19, by attrition status and baseline characteristics, Kinshasa. (DOCX)

S2 Table Percent distribution and mean age of first-time mothers age 20–24, by attrition status and baseline characteristics, Kinshasa. (DOCX)

S3 Table Percent distribution of first-time mothers age 15–19, by components of the Momentum intervention, marital status, and study arm, Kinshasa. (DOCX)

S4 Table Percent distribution of first-time mothers age 20–24, by components of the Momentum intervention, marital status, and study arm, Kinshasa. (DOCX)

S5 Table Mean outcomes at baseline among first-time mothers age 15–19, by attrition status, marital status, and study arm, Kinshasa. (DOCX)

S6 Table Mean outcomes at baseline among first-time mothers age 20–24, by attrition status, marital status, and study arm, Kinshasa. (DOCX)

S7 Table Average treatment effects for family planning behavioral outcomes by age group, marital status, and type of model (intent-to-treat analysis), first-time mothers age 15–24, Kinshasa. (DOCX)
