# Supplementary material for: Effects of the Momentum project on postpartum family planning norms and behaviors among married and unmarried adolescent and young first-time mothers in Kinshasa: A quasi-experimental study
Source: PLoS One. 2024 Mar 28;19(3):e0300342. doi: 10.1371/journal.pone.0300342 (PMC10977807; doi:10.1371/journal.pone.0300342)
Supplement: S1 Table — (DOCX) [file pone.0300342.s001.docx]

**S1 Table. Percent distribution and mean age of first-time mothers age 15-19 by attrition status and baseline characteristics, Kinshasa**

|  | **Never Married** | | | | | | |  | **Ever Married/Engaged** | | | | | | |
| --- | --- | --- | --- | --- | --- | --- | --- | --- | --- | --- | --- | --- | --- | --- | --- |
|  | **Comparison** | | |  | **Intervention** | | |  | **Comparison** | | |  | **Intervention** | | |
| **Baseline Characteristics** | **LTFU** | **Retained Cases** | **p-value** |  | **LTFU** | **Retained Cases** | **p-value** |  | **LTFU** | **Retained Cases** | **p-value** |  | **LTFU** | **Retained Cases** | **p-value** |
| Mean age (SD) | 17.6 (1.7) | 17.6 (1.4) | 0.780 |  | 17.6 (1.5) | 17.7 (1.6) | 0.803 |  | 18.4 (1.4) | 18.0 (1.4) | 0.021 |  | 18.3 1.4) | 18.2 (1.4) | 0.551 |
| FTM's years of schooling |  |  | 0.262 |  |  |  | 0.624 |  |  |  | 0.713 |  |  |  | 0.169 |
| Low | 70.2 | 61.5 |  |  | 64.6 | 60.8 |  |  | 51.1 | 48.9 |  |  | 55.9 | 48.7 |  |
| High | 29.8 | 38.5 |  |  | 35.4 | 39.2 |  |  | 48.9 | 51.1 |  |  | 44.1 | 51.3 |  |
| Both parents have secondary or higher education |  |  | 0.488 |  |  |  | 0.274 |  |  |  | 0.841 |  |  |  | 0.310 |
| No | 29.8 | 24.9 |  |  | 22.9 | 16.3 |  |  | 18.1 | 19.0 |  |  | 24.6 | 20.2 |  |
| Yes | 78.2 | 75.1 |  |  | 77.1 | 83.7 |  |  | 81.9 | 81.0 |  |  | 75..4 | 79.8 |  |
| Watched TV at least once a week |  |  | 0.652 |  |  |  | 0.831 |  |  |  | 0.812 |  |  |  | 0.029 |
| No | 34.0 | 37.6 |  |  | 43.8 | 45.4 |  |  | 35.1 | 36.4 |  |  | 48.3 | 37.0 |  |
| Yes | 66.0 | 62.4 |  |  | 56.3 | 54.6 |  |  | 64.9 | 63.6 |  |  | 51.7 | 63.0 |  |
| Ethnicity |  |  | 0.850 |  |  |  | 0.451 |  |  |  | 0.202 |  |  |  | 0.304 |
| Bakongo | 21.3 | 23.9 |  |  | 35.5 | 34.9 |  |  | 33.0 | 29.3 |  |  | 51.7 | 44.1 |  |
| Bas Kasai & Kwilu-Kwango | 18.9 | 42.4 |  |  | 22.9 | 25.8 |  |  | 29.8 | 33.3 |  |  | 12.7 | 18.1 |  |
| Kasai/Katana/Tanganyika | 10.6 | 10.2 |  |  | 20.8 | 12.5 |  |  | 22.3 | 15.3 |  |  | 18.6 | 16.8 |  |
| Other | 19.2 | 23.4 |  |  | 20.8 | 26.8 |  |  | 14.9 | 22.1 |  |  | 17.0 | 21.0 |  |
| Household wealth |  |  | 0.206 |  |  |  | 0.207 |  |  |  | 0.624 |  |  |  | 0.172 |
| Low | 27.7 | 37.4 |  |  | 53.2 | 39.2 |  |  | 38.3 | 32.9 |  |  | 49.1 | 40.5 |  |
| Medium | 42.5 | 29.6 |  |  | 23.4 | 32.6 |  |  | 33.0 | 35.4 |  |  | 34.5 | 36.4 |  |
| High | 29.8 | 33.0 |  |  | 23.4 | 28.2 |  |  | 27.7 | 31.7 |  |  | 16.4 | 23.1 |  |
| Attrition Rate (%) | 18.7 | | |  | 18.7 | | |  | 22.7 | | |  | 23.4 | | |
| N | 47 | 205 |  |  | 48 | 209 |  |  | 94 | 321 |  |  | 118 | 386 |  |

FTM – first-time mother

Notes: P-values pertain to the differences in baseline characteristics between retained cases and those lost to follow-up.

SD Standard deviation
