# Supplementary material for: Effects of the Momentum project on postpartum family planning norms and behaviors among married and unmarried adolescent and young first-time mothers in Kinshasa: A quasi-experimental study
Source: PLoS One. 2024 Mar 28;19(3):e0300342. doi: 10.1371/journal.pone.0300342 (PMC10977807; doi:10.1371/journal.pone.0300342)
Supplement: S2 Table — (DOCX) [file pone.0300342.s002.docx]

**S2 Table. Percent distribution and mean age of first-time mothers age 20-24 by attrition status and baseline characteristics, Kinshasa**

|  | **Never Married** | | | | | | |  | **Ever Married/Engaged** | | | | | | | | |  |  |  |  |  |
| --- | --- | --- | --- | --- | --- | --- | --- | --- | --- | --- | --- | --- | --- | --- | --- | --- | --- | --- | --- | --- | --- | --- |
|  | **Comparison** | | |  | **Intervention** | | |  | **Comparison** | | |  | **Intervention** | | | | |  |  |  |  |  |
| **Baseline Characteristics** | **LTFU** | **Retained Cases** | **p-value** |  | **LTFU** | **Retained Cases** | **p-value** |  | **LTFU** | **Retained Cases** | **p-value** |  | **LTFU** | **Retained Cases** | | **p-value** | |  |  |  |  |  |
| Mean age (SD) | 22.3 (1.1) | 22.2 (1.0) | 0.749 |  | 22.1 (1.2) | 22.6 (1.2) | 0.125 |  | 22.8 (1.1) | 22.7 (1.2) | 0.279 |  | 22.4 (1.17) | | 22.7 (1.2) | | 0.046 | |  |  |  |  |
|  |  |  |  |  |  |  |  |  |  |  |  |  |  | |  | |  | |  |  |  |  |
| FTM's years of schooling |  |  | 0.027 |  |  |  | 0.437 |  |  |  | 0.716 |  |  |  | | 0.645 | |  |  |  |  |  |
| Low | [55.6] | 28.7 |  |  | [25.0] | 35.1 |  |  | 20.2 | 22.1 |  |  | 26.6 | 24.1 | |  | |  |  |  |  |  |
| High | [44.4] | 71.3 |  |  | [75.0] | 64.9 |  |  | 79.8 | 77.9 |  |  | 73.4 | 75.9 | |  | |  |  |  |  |  |
| Both parents have secondary or higher education |  |  | 0.246 |  |  |  | 0.478 |  |  |  | 0.337 |  |  |  | | 0.735 | |  |  |  |  |  |
| No | [38.9] | 25.5 |  |  | [18.8] | 27.3 |  |  | 22.6 | 18.0 |  |  | 20.2 | 22.0 | |  | |  |  |  |  |  |
| Yes | [61.1] | 74.5 |  |  | [81.3] | 72.7 |  |  | 77.4 | 82.0 |  |  | 79.8 | 78.0 | |  | |  |  |  |  |  |
| Watched TV at least once a week |  |  | 0.962 |  |  |  | 0.113 |  |  |  | 0.947 |  |  |  | | 0.846 | |  |  |  |  |  |
| No | [38.9] | 38.3 |  |  | [56.3] | 35.1 |  |  | 35.7 | 36.1 |  |  | 35.4 | 34.3 | |  | |  |  |  |  |  |
| Yes | [61.1] | 61.7 |  |  | [43.7] | 64.9 |  |  | 64.3 | 63.9 |  |  | 64.6 | 65.7 | |  | |  |  |  |  |  |
| Ethnicity |  |  | 0.648 |  |  |  | 0.023 |  |  |  | 0.841 |  |  |  | | 0.632 | |  |  |  |  |  |
| Bakongo | [16.7] | 28.7 |  |  | [68.8 | 31.2 |  |  | 33.3 | 37.0 |  |  | 53.2 | 47.9 | |  | |  |  |  |  |  |
| Bas Kasai & Kwilu-Kwango | [38.8] | 38.3 |  |  | [6.2] | 32.5 |  |  | 36.9 | 32.1 |  |  | 12.6 | 18.5 | |  | |  |  |  |  |  |
| Kasai/Katana/Tanganyika | [16.7] | 9.6 |  |  | [12.5] | 9.0 |  |  | 16.7 | 16.3 |  |  | 15.2 | 13.7 | |  | |  |  |  |  |  |
| Other | [27.8] | 23.4 |  |  | [12.5] | 27.3 |  |  | 13.1 | 14.6 |  |  | 19.0 | 19.9 | |  | |  |  |  |  |  |
| Household wealth |  |  | 0.924 |  |  |  | 0.126 |  |  |  | 0.179 |  |  |  | | 0.028 | |  |  |  |  |  |
| Low | [33.3] | 30.8 |  |  | [31.2] | 32.0 |  |  | 31.3 | 22.7 |  |  | 48.1 | 33.0 | |  | |  |  |  |  |  |
| Medium | [33.4] | 30.9 |  |  | [50.0] | 26.7 |  |  | 27.7 | 36.2 |  |  | 30.4 | 33.0 | |  | |  |  |  |  |  |
| High | [33.3] | 38.3 |  |  | [18.8] | 41.3 |  |  | 41.0 | 41.1 |  |  | 21.5 | 34.0 | |  | |  |  |  |  |  |
| Attrition Rate (%) | 16.1 | | |  | 17.2 | | |  | 19.4 | | |  | 21.6 | | | | |  | |  | 21.6 |  |
| N | [18] | 94 |  |  | [16] | 77 |  |  | 84 | 349 |  |  | 79 | 286 | |  | |  |  |  |  |  |

FTM – first-time mother

Notes: P-values pertain to the differences in baseline characteristics between retained cases and those lost to follow-up.

SD Standard deviation

[ ] Small number of cases
