# Supplementary material for: Effects of the Momentum project on postpartum family planning norms and behaviors among married and unmarried adolescent and young first-time mothers in Kinshasa: A quasi-experimental study
Source: PLoS One. 2024 Mar 28;19(3):e0300342. doi: 10.1371/journal.pone.0300342 (PMC10977807; doi:10.1371/journal.pone.0300342)
Supplement: S3 Table — (DOCX) [file pone.0300342.s003.docx]

**S3 Table. Percent distribution of first-time mothers age 15-19 by components of the Momentum intervention, marital status, and study arm, Kinshasa**

|  | **Never Married** | | | |  | **Ever Married/Engaged** | | | |
| --- | --- | --- | --- | --- | --- | --- | --- | --- | --- |
|  | **Comparison** | | **Intervention** | |  | **Comparison** | | **Intervention** | |
| **Intervention Component** | **N** | **%** | **N** | **%** |  | **N** | **%** | **N** | **%** |
| A. Home Visits |  |  |  |  |  |  |  |  |  |
| Received prenatal home visit by Momentum nursing student |  |  |  |  |  |  |  |  |  |
| No | 186 | 100.0 | 41 | 23.8 |  | 257 | 100.0 | 80 | 25.6 |
| Yes | 0 | 0.0 | 131 | 76.2 |  | 0 | 0.0 | 233 | 74.4 |
| Received postnatal home visit by Momentum nursing student |  |  |  |  |  |  |  |  |  |
| No | 186 | 100.0 | 76 | 44.2 |  | 257 | 100.0 | 125 | 39.9 |
| Yes | 0 | 0.0 | 96 | 55.8 |  | 0 | 0.0 | 188 | 60.6 |
| Received home visit by Momentum nursing student |  |  |  |  |  |  |  |  |  |
| No | 186 | 100.0 | 33 | 19.2 |  | 257 | 100.0 | 62 | 19.8 |
| Prenatal visit only | 0 | 0.0 | 43 | 25.0 |  | 0 | 0.0 | 63 | 20.1 |
| Postnatal visit only | 0 | 0.0 | 8 | 4.6 |  | 0 | 0.0 | 18 | 5.8 |
| Both prenatal and postnatal visit | 0 | 0.0 | 88 | 51.2 |  | 0 | 0.0 | 170 | 54.3 |
| Total number of home visits (prenatal and postnatal) |  |  |  |  |  |  |  |  |  |
| None | 186 | 100.0 | 33 | 19.2 |  | 257 | 100.0 | 62 | 19.8 |
| 1-3 | 0 | 0.0 | 63 | 36.6 |  | 0 | 0.0 | 99 | 31.6 |
| 4-6 | 0 | 0.0 | 47 | 27.3 |  | 0 | 0.0 | 98 | 31.3 |
| 7+ | 0 | 0.0 | 29 | 16.9 |  | 0 | 0.0 | 54 | 17.3 |
|  |  |  |  |  |  |  |  |  |  |
| B. Group Education Sessions |  |  |  |  |  |  |  |  |  |
| Participated in group education sessions |  |  |  |  |  |  |  |  |  |
| No | 186 | 100.0 | 82 | 47.7 |  | 255 | 99.2 | 153 | 48.9 |
| Prenatal period only | 0 | 0.0 | 30 | 13.4 |  | 2 | 0.8 | 53 | 16.9 |
| Postnatal period only | 0 | 0.0 | 37 | 21.5 |  | 0 | 0.0 | 65 | 20.8 |
| Both periods | 0 | 0.0 | 23 | 17.4 |  | 0 | 0.0 | 42 | 13.4 |
| No. of group education sessions attended |  |  |  |  |  |  |  |  |  |
| None | 186 | 100.0 | 82 | 47.7 |  | 255 | 99.2 | 154 | 49.2 |
| 1-2 | 0 | 0.0 | 44 | 25.6 |  | 1 | 0.4 | 83 | 26.5 |
| 3-4 | 0 | 0.0 | 25 | 14.5 |  | 0 | 0.0 | 49 | 15.7 |
| 5+ | 0 | 0.0 | 16 | 9.3 |  | 1 | 0.4 | 21 | 6.7 |
| Do not know | 0 | 0.0 | 5 | 2.9 |  | 0 | 0.0 | 6 | 1.9 |
| Total contacts (home visits and group education) |  |  |  |  |  |  |  |  |  |
| None | 186 | 100.0 | 25 | 14.5 |  | 255 | 99.2 | 52 | 16.7 |
| 1-3 | 0 | 0.0 | 45 | 26.2 |  | 1 | 0.8 | 72 | 23.0 |
| 4-6 | 0 | 0.0 | 47 | 27.3 |  | 1 | 0.8 | 92 | 29.4 |
| 7-9 | 0 | 0.0 | 26 | 15.1 |  | 0 | 0.0 | 52 | 16.6 |
| 10+ | 0 | 0.0 | 29 | 16.9 |  | 0 | 0.0 | 45 | 14.4 |
|  |  |  |  |  |  |  |  |  |  |
| Level of exposure to Momentum |  |  |  |  |  |  |  |  |  |
| None | 186 | 100.0 | 25 | 14.5 |  | 255 | 99.2 | 52 | 16.6 |
| Partial (home visits or group education) | 0 | 0.0 | 65 | 37.8 |  | 2 | 0.8 | 111 | 35.5 |
| Full (both home visits and group education) | 0 | 0.0 | 82 | 47.7 |  | 0 | 0.0 | 150 | 47.9 |
|  |  | |  | |  |  | |  | |
| N | 186 | | 172 | |  | 257 | | 313 | |

Notes: Data pertain to first-time mothers who were interviewed in both the baseline and follow-up surveys.
