# Supplementary material for: Effects of the Momentum project on postpartum family planning norms and behaviors among married and unmarried adolescent and young first-time mothers in Kinshasa: A quasi-experimental study
Source: PLoS One. 2024 Mar 28;19(3):e0300342. doi: 10.1371/journal.pone.0300342 (PMC10977807; doi:10.1371/journal.pone.0300342)
Supplement: S4 Table — (DOCX) [file pone.0300342.s004.docx]

**S4** **Table. Percent distribution of first-time mothers age 20-24 by components of the Momentum intervention, marital status, and study arm, Kinshasa**

|  | **Never Married** | | | |  | **Ever Married/Engaged** | | | |
| --- | --- | --- | --- | --- | --- | --- | --- | --- | --- |
|  | **Comparison** | | **Intervention** | |  | **Comparison** | | **Intervention** | |
| **Intervention Component** | **N** | **%** | **N** | **%** |  | **N** | **%** | **N** | **%** |
| A. Home Visits |  |  |  |  |  |  |  |  |  |
| Received prenatal home visit by Momentum nursing student |  |  |  |  |  |  |  |  |  |
| No | 113 | 100.0 | 26 | 13.0 |  | 412 | 99.8 | 79 | 22.1 |
| Yes | 0 | 0.0 | 87 | 77.0 |  | 1 | 0.2 | 278 | 77.9 |
| Received postnatal home visit by Momentum nursing student |  |  |  |  |  |  |  |  |  |
| No | 113 | 100.0 | 42 | 37.2 |  | 413 | 100.0 | 135 | 37.8 |
| Yes | 0 | 0.0 | 71 | 62.8 |  | 0 | 0.0 | 222 | 62.2 |
| Received home visit by Momentum nursing student |  |  |  |  |  |  |  |  |  |
| No | 113 | 100.0 | 23 | 20.4 |  | 412 | 99.8 | 63 | 17.7 |
| Prenatal visit only | 0 | 0.0 | 19 | 16.8 |  | 1 | 0.2 | 72 | 20.2 |
| Postnatal visit only | 0 | 0.0 | 3 | 2.6 |  | 0 | 0.0 | 16 | 4.4 |
| Both prenatal and postnatal visit | 0 | 0.0 | 68 | 60.2 |  | 0 | 0.0 | 206 | 57.7 |
| Total number of home visits (prenatal and postnatal) |  |  |  |  |  |  |  |  |  |
| None | 113 | 100.0 | 23 | 20.3 |  | 412 | 99.8 | 63 | 17.7 |
| 1-3 | 0 | 0.0 | 36 | 31.9 |  | 1 | 0.2 | 115 | 32.2 |
| 4-6 | 0 | 0.0 | 28 | 24.8 |  | 0 | 0.0 | 121 | 33.9 |
| 7+ | 0 | 0.0 | 26 | 23.0 |  | 0 | 0.0 | 58 | 16.2 |
|  |  |  |  |  |  |  |  |  |  |
| B. Group Education Sessions |  |  |  |  |  |  |  |  |  |
| Participated in group education sessions |  |  |  |  |  |  |  |  |  |
| No | 113 | 100.0 | 50 | 44.2 |  | 408 | 98.8 | 156 | 43.7 |
| Prenatal period only | 0 | 0.0 | 16 | 14.2 |  | 5 | 1.2 | 58 | 16.3 |
| Postnatal period only | 0 | 0.0 | 30 | 26.6 |  | 0 | 0.0 | 70 | 19.6 |
| Both periods | 0 | 0.0 | 17 | 15.0 |  | 0 | 0.0 | 73 | 20.4 |
| No. of group education sessions attended |  |  |  |  |  |  |  |  |  |
| None | 113 | 100.0 | 50 | 44.2 |  | 408 | 98.8 | 156 | 43.7 |
| 1-2 | 0 | 0 | 27 | 23.9 |  | 4 | 1.0 | 99 | 27.7 |
| 3-4 | 0 | 0 | 14 | 12.4 |  | 0 | 0.0 | 59 | 16.5 |
| 5+ | 0 | 0 | 15 | 13.3 |  | 1 | 0.2 | 33 | 9.2 |
| Do not know | 0 | 0 | 7 | 6.2 |  | 0 | 0.0 | 10 | 2.8 |
| Total contacts (home visits and group education) |  |  |  |  |  |  |  |  |  |
| None | 113 | 100.0 | 19 | 16.8 |  | 407 | 98.6 | 52 | 14.6 |
| 1-3 | 0 | 0.0 | 28 | 24.8 |  | 5 | 1.2 | 76 | 21.3 |
| 4-6 | 0 | 0.0 | 25 | 22.1 |  | 1 | 0.2 | 105 | 29.4 |
| 7-9 | 0 | 0.0 | 15 | 13.3 |  | 0 | 0.0 | 69 | 19.3 |
| 10+ | 0 | 0.0 | 26 | 23.0 |  | 0 | 0.0 | 55 | 15.4 |
|  |  |  |  |  |  |  |  |  |  |
| Level of exposure to Momentum |  |  |  |  |  |  |  |  |  |
| None | 113 | 100.0 | 19 | 16.8 |  | 407 | 98.6 | 52 | 14.6 |
| Partial (home visits or group education) | 0 | 0.0 | 35 | 31.0 |  | 6 | 1.4 | 115 | 32.2 |
| Full (both home visits and group education) | 0 | 0.0 | 59 | 52.2 |  | 0 | 0.0 | 190 | 53.2 |
|  |  | |  | |  |  | |  | |
| N | 113 | | 113 | |  | 413 | | 357 | |

Notes: Data pertain to first-time mothers who were interviewed in both the baseline and follow-up surveys.
