# Supplementary material for: Effects of the Momentum project on postpartum family planning norms and behaviors among married and unmarried adolescent and young first-time mothers in Kinshasa: A quasi-experimental study
Source: PLoS One. 2024 Mar 28;19(3):e0300342. doi: 10.1371/journal.pone.0300342 (PMC10977807; doi:10.1371/journal.pone.0300342)
Supplement: S5 Table — (DOCX) [file pone.0300342.s005.docx]

**S5 Table.** **Mean outcomes at baseline among first-time mothers age 15-19 by attrition status, marital status, and study arm, Kinshasa**

|  | **Never Married** | | | | | | |  | **Ever Married/Engaged** | | | | | | |
| --- | --- | --- | --- | --- | --- | --- | --- | --- | --- | --- | --- | --- | --- | --- | --- |
|  | **Comparison** | | |  | **Intervention** | | |  | **Comparison** | | |  | **Intervention** | | |
| **Outcome** | **LTFU** | **Retained Cases** | **p-value** |  | **LTFU** | **Retained Cases** | **p-value** |  | **LTFU** | **Retained Cases** | **p-value** |  | **LTFU** | **Retained Cases** | **p-value** |
| *Normative expectations* |  |  |  |  |  |  |  |  |  |  |  |  |  |  |  |
| Perceived that significant others believe the FTM ought to discuss PPFP (%) | 66.7 | 68.4 | 0.815 |  | 63.8 | 77.1 | 0.060 |  | 70.3 | 68.4 | 0.690 |  | 62.8 | 60.7 | 0.725 |
| Perceived that significant others believe the FTM ought to use PPFP (%) | 72.9 | 71.3 | 0.823 |  | 70.2 | 73.7 | 0.633 |  | 72.0 | 39.4 | 0.590 |  | 64.9 | 62.0 | 0.610 |
| *Descriptive norms* |  |  |  |  |  |  |  |  |  |  |  |  |  |  |  |
| Believed most of FTMs 15-24 in community discuss PPFP with husband/partner before baby's birth (%) | 6.3 | 8.1 | 0.662 |  | 14.9 | 15.6 | 0.903 |  | 6.8 | 8.8 | 0.486 |  | 11.7 | 12.1 | 0.907 |
| Believed most of FTMs 15-24 in community use PPFP (%) | 6.3 | 10.0 | 0.417 |  | 17.0 | 16.1 | 0.878 |  | 9.3 | 8.8 | 0.864 |  | 8.5 | 12.8 | 0.261 |
| *Injunctive norms* |  |  |  |  |  |  |  |  |  |  |  |  |  |  |  |
| Perceived that community members will say good things about women who use PPFP (%) | 25.0 | 25.8 | 0.905 |  | 34.1 | 42.9 | 0.266 |  | 20.3 | 25.6 | 0.241 |  | 33.0 | 35.2 | 0.691 |
| *Personal agency* |  |  |  |  |  |  |  |  |  |  |  |  |  |  |  |
| Mean personal agency score (SD) | 18.3 (5.7) | 18.6  (5.4) | 0.725 |  | 19.5 (4.9) | 19.0  (4.9) | 0.518 |  | 19.6 (5.8) | 19.4  (5.1) | 0.615 |  | 18.0 (5.2) | 18.0  (5.1) | 0.983 |
| N | 48 | 209 |  |  | 47 | 205 |  |  | 118 | 386 |  |  | 94 | 321 |  |

FTM – first-time mother; FP – family planning; PPFP – postpartum family planning
