# Supplementary material for: Effects of the Momentum project on postpartum family planning norms and behaviors among married and unmarried adolescent and young first-time mothers in Kinshasa: A quasi-experimental study
Source: PLoS One. 2024 Mar 28;19(3):e0300342. doi: 10.1371/journal.pone.0300342 (PMC10977807; doi:10.1371/journal.pone.0300342)
Supplement: S6 Table — (DOCX) [file pone.0300342.s006.docx]

**S6 Table.** **Mean outcomes at baseline among first-time mothers age 20-24 by attrition status, marital status, and study arm, Kinshasa**

|  | **Never Married** | | | | | | |  | **Ever Married/Engaged** | | | | | | |
| --- | --- | --- | --- | --- | --- | --- | --- | --- | --- | --- | --- | --- | --- | --- | --- |
|  | **Comparison** | | |  | **Intervention** | | |  | **Comparison** | | |  | **Intervention** | | |
| **Outcome** | **LTFU** | **Retained Cases** | **p-value** |  | **LTFU** | **Retained Cases** | **p-value** |  | **LTFU** | **Retained Cases** | **p-value** |  | **LTFU** | **Retained Cases** | **p-value** |
| *Normative expectations* |  |  |  |  |  |  |  |  |  |  |  |  |  |  |  |
| Perceived that significant others believe the FTM ought to discuss PPFP (%) | [66.7] | 75.5 | 0.436 |  | [68.8] | 66.2 | 0.848 |  | 63.1 | 68.8 | 0.320 |  | 72.2 | 68.5 | 0.538 |
| Perceived that significant others believe the FTM ought to use PPFP (%) | [72.2] | 77.7 | 0.620 |  | [81.3] | 74.0 | 0.547 |  | 61.9 | 63.3 | 0.809 |  | 72.2 | 68.9 | 0.577 |
| *Descriptive norms* |  |  |  |  |  |  |  |  |  |  |  |  |  |  |  |
| Believed most of FTMs 15-24 in community discuss PPFP with husband/partner before baby's birth (%) | [11.1] | 19.1 | 0.419 |  | [12.5] | 11.7 | 0.928 |  | 6.0 | 10.6 | 0.197 |  | 8.9 | 10.5 | 0.672 |
| Believed most of FTMs 15-24 in community use PPFP (%) | [16.7] | 18.1 | 0.887 |  | [18.8] | 10.4 | 0.352 |  | 6.0 | 14.0 | 0.044 |  | 10.1 | 9.8 | 0.930 |
| *Injunctive norms* |  |  |  |  |  |  |  |  |  |  |  |  |  |  |  |
| Perceived that community members will say good things about women who use PPFP (%) | [72.2] | 40.4 | 0.013 |  | [37.5] | 27.3 | 0.418 |  | 32.1 | 41.0 | 0.137 |  | 24.1 | 25.2 | 0.839 |
| *Personal agency* |  |  |  |  |  |  |  |  |  |  |  |  |  |  |  |
| Mean personal agency score | [18.0] (5.6) | 19.7  (4.9) | 0.189 |  | [20.5] (6.3) | 19.9  (5.5) | 0.711 |  | 18.2 (5.6) | 18.9 (5.1) | 0.244 |  | 18.6 (5.5) | 19.3  (5.3) | 0.257 |
|  |  |  |  |  |  |  |  |  |  |  |  |  |  |  |  |
| N | [18] | 94 |  |  | [16] | 77 |  |  | 84 | 349 |  |  | 79 | 286 |  |

FTM – first-time mother; PPFP – postpartum family planning

[ ] Small number of cases
