# Supplementary material for: Effects of the Momentum project on postpartum family planning norms and behaviors among married and unmarried adolescent and young first-time mothers in Kinshasa: A quasi-experimental study
Source: PLoS One. 2024 Mar 28;19(3):e0300342. doi: 10.1371/journal.pone.0300342 (PMC10977807; doi:10.1371/journal.pone.0300342)
Supplement: S7 Table — (DOCX) [file pone.0300342.s007.docx]

S7 Table. Average treatment effects for family planning behavioral outcomes by age group, marital status, and type of model (intent-to-treat analysis), first-time mothers age 15-24, Kinshasa

|  | **Age 15-19** | | | | | | |  | **Age 20-24** | | | | | | |
| --- | --- | --- | --- | --- | --- | --- | --- | --- | --- | --- | --- | --- | --- | --- | --- |
|  | **Ever Married** | | |  | **Never Married** | | |  | **Ever Married** | | |  | **Never Married** | | |
| **Outcome** | **ATE** | **p-value** | **95% CI** |  | **ATE** | **p-value** | **95% CI** |  | **ATE** | **p-value** | **95% CI** |  | **ATE** | **p-value** | **95% CI** |
| In early postpartum period,  discussed FP with male partner |  |  |  |  |  |  |  |  |  |  |  |  |  |  |  |
| IPW | 0.179 | <0.001 | (0.098, 0.261) |  | 0.131 | 0.011 | (0.029, 0.232) |  | 0.233 | <0.001 | (0.164, 0.302) |  | 0.241 | <0.001 | (0.121, 0.362) |
| AIPW | 0.181 | <0.001 | (0.099, 0.263) |  | 0.133 | 0.009 | (0.033, 0.233) |  | 0.232 | <0.001 | (0.163, 0.301) |  | 0.241 | <0.001 | (0.120, 0.361) |
| PSM | 0.205 | <0.001 | (0.106, 0.304) |  | 0.098 | 0.095 | (-0.017, 0.213) |  | 0.220 | <0.001 | (0.140, 0.301) |  | 0.180 | 0.026 | (0.022, 0.338) |
| In early postpartum period,  discussed FP with health worker |  |  |  |  |  |  |  |  |  |  |  |  |  |  |  |
| IPW | 0.260 | <0.001 | (0.179, 0.341) |  | 0.128 | 0.015 | (0.025, 0.231) |  | 0.275 | <0.001 | (0.208, 0.343) |  | 0.306 | <0.001 | (0.185, 0.427) |
| AIPW | 0.259 | <0.001 | (0.178, 0.339) |  | 0.136 | 0.009 | (0.034, 0.238) |  | 0.275 | <0.001 | (0.208, 0.343) |  | 0.302 | <0.001 | (0.181, 0.423) |
| PSM | 0.257 | <0.001 | (0.161, 0.352) |  | 0.123 | 0.046 | (0.002, 0.244) |  | 0.268 | <0.001 | (0.192, 0.345) |  | 0.302 | <0.001 | (0.153, 0.449) |
| In early postpartum period,  obtained a contraceptive method |  |  |  |  |  |  |  |  |  |  |  |  |  |  |  |
| IPW | 0.190 | <0.001 | (0.123, 0.257) |  | 0.061 | 0.139 | (-0.020, 0.143) |  | 0.097 | 0.002 | (0.035, 0.158) |  | 0.220 | <0.001 | (0.104, 0.336) |
| AIPW | 0.189 | <0.001 | (0.122, 0.256) |  | 0.064 | 0.118 | (-0.016, 0.145) |  | 0.099 | 0.002 | (0.037, 0.160) |  | 0.215 | <0.001 | (0.100, 0.331) |
| PSM | 0.214 | <0.001 | (0.140, 0.289) |  | 0.037 | 0.445 | (-0.059, 0.134) |  | 0.117 | 0.002 | (0.042, 0.193) |  | 0.228 | 0.004 | (0.071, 0.385) |
| PPFP use (a) |  |  |  |  |  |  |  |  |  |  |  |  |  |  |  |
| IPW | 0.114 | 0.017 | (0.020, 0.208) |  | 0.251 | <0.001 | (0.122, 0.380) |  | 0.113 | 0.004 | (0.036, 0.190) |  | 0.098 | 0.193 | (-0.050, 0.245) |
| AIPW | 0.124 | 0.010 | (0.030, 0.218) |  | 0.244 | <0.001 | (0.116, 0.372) |  | 0.112 | 0.004 | (0.036, 0.189) |  | 0.091 | 0.222 | (-0.055, 0.237) |
| PSM | 0.111 | 0.058 | (-0.002, 0.224) |  | 0.231 | 0.004 | (0.072, 0.391) |  | 0.083 | 0.074 | (-0.008, 0.173) |  | -0.003 | 0.970 | (-0.163, 0.157) |
| N | 570 | | |  | 358 | | |  | 770 | | |  | 226 | | |

Notes: Regressions control for the following baseline characteristics of the FTM: single years of age, years of schooling, household wealth, ethnicity, parental education, weekly television exposure, gender equity score, and power score. FP – family planning, PPFP – postpartum family planning

1. Restricted to women who resumed sexual activity 0-11 months after childbirth/pregnancy loss. Ns are as follows: Ever married 15-19 = 439; Never married 15-19 = 229; Ever Married 20-24 = 661; Never married 20-24 = 165.
